# Grinding and Fractionation during Distillation Alter Hemp Essential Oil Profile and Its Antimicrobial Activity

**DOI:** 10.3390/molecules25173943

**Published:** 2020-08-28

**Authors:** Valtcho D. Zheljazkov, Vladimir Sikora, Ivanka B. Semerdjieva, Miroslava Kačániová, Tess Astatkie, Ivayla Dincheva

**Affiliations:** 1 Crop and Soil Science Department, 3050 SW Campus Way, Oregon State University, Corvallis, OR 97331, USA; 2 Institute for Field and Vegetable Crops, Alternative Crops and Organic Production Department, Maksima Gorkog 30, 21000 Novi Sad, Serbia; vladimir.sikora@ifvcns.ns.ac.rs; 3 Department of Botany and Agrometeorology, Faculty of Agronomy, Agricultural University, 4000 Plovdiv, Bulgaria; v_semerdjieva@abv.bg; 4 Department of Fruit Science, Viticulture and Enology, Faculty of Horticulture and Landscape Engineering, Tr. A. Hlinku 2, Slovak University of Agriculture in Nitra, 949 76 Nitra, Slovak Republic; kacaniova.miroslava@gmail.com; 5 Department of Bioenergetics and Food Analysis, Institution of Food Technology and Nutrition, University of Rzeszow, Cwiklinskiej 1, 35-601 Rzeszow, Poland; 6 Department of Engineering, Faculty of Agriculture, Dalhousie University, Truro, NS B2N 5E3, Canada; astatkie@dal.ca; 7 Plant Genetic Research Group, Agrobioinstitute, Agricultural Academy, 8 “Dragan Tsankov” Blvd, 1164 Sofia, Bulgaria; ivadincheva@yahoo.com

**Keywords:** *Cannabis sativa*, volatile oil, cannabinoids, cannabidiol, *δ*9-tetrahydrocannabinol, dronabinol, monoterpenes, sesquiterpenes, bioactivity

## Abstract

The hypothesis of this study was that we can modify the essential oil (EO) profile of hemp (*Cannabis sativa* L.) and obtain fractions with differential composition and antimicrobial activity. Therefore, the objective was to evaluate the effects of grinding of hemp biomass before EO extraction and fractionation during distillation on EO profile and antimicrobial activity. The study generated a several EO fractions with a diversity of chemical profile and antimicrobial activity. The highest concentrations of *β*-pinene and myrcene in the EO can be obtained in the 5–10 min distillation time (DT) of ground material or in the 80–120 min DT of nonground material. High *δ*-3-carene and limonene EO can be obtained from 0–5 min DT fraction of nonground material. High eucalyptol EO can be sampled either in the 0–5 min DT of the ground material or in the 80–120 min of nonground material. Overall, the highest concentrations of *β*-caryophyllene, *α*-(*E*)-bergamotene, (*Z*)-*β*-farnesene, *α*-humulene, caryophyllenyl alcohol, germacrene D-4-ol, spathulenol, caryophyllene oxide, humulene epoxide 2, *β*-bisabolol, *α*-bisabolol, sesquiterpenes, and cannabidiol (CBD) can be obtained when EO is sampled in the 80–120 min DT and the material is nonground. Monoterpenes in the hemp EO can be increased twofold to 85% by grinding the material prior to distillation and collecting the EO in the first 10 min. However, grinding resulted in a slight but significant decrease in the CBD concentration of the EO. CBD-rich oil can be produced by collecting at 120–180 min DT. Different EO fractions had differential antimicrobial activity. The highest antimicrobial activity of EO fraction was found against *Staphylococcus aureus* subsp. *aureus*. THC-free EO can be obtained if the EO distillation is limited to 120 min. The results can be utilized by the hemp processing industry and by companies developing new hemp EO-infused products, including perfumery, cosmetics, dietary supplements, food, and pharmaceutical industries.

## 1. Introduction

Hemp (*Cannabis sativa* L.) has been and continues to be a controversial crop, although it is one of the oldest crops utilized by humans [1,2]. Hemp has multiple utilizations; traditionally it has been used as a fiber and oilseed crop [3,4] due to the high content of high-quality durable fiber in the stem [5] and its seed that is high in both protein and fatty acids (fixed) oil [6,7]. Hemp has also a long history as being used as a psychoactive plant in various religions, other spiritual rituals or as recreational/hedonic [5,8].

The pharmacological power of hemp depends on the content of Δ9-tetrahydrocannabinolic acid (THCA) and cannabidiolic acid (CBDA) [9,10], but also on terpenes entourage effects [2,11,12]. Other major cannabinoids include cannabinolic acid (CBNA), cannabigerolic acid (CBGA), cannabichromenic acid (CBCA) and cannabinodiolic acid (CBNDA) [13,14]. All these chemicals are also found in industrial hemp (with legal requirement of THC content < 0.3%). The concentration of these natural products varies depending on plant genotype (cultivar, variety), the plant growth stage, growth conditions and fertilization, and plant part [13,15,16] and also on the postharvest heating treatment [17].

Hemp production for high-value chemicals has recently amplified beyond anyone’s expectations [18], driven largely by the quickly established market for products infused with hemp or with hemp extracts such as ”CBD oil”, which include terpenes. The current environment of rigorous new product development with hemp extracts expanded the need and demand for hemp essential oil (EO) with differential chemical profile, aroma, and bioactivity [19,20,21,22]. This can be achieved by either growing a large number of varieties and rely on their genetic and phytochemical differences, or by fractionating EO from a single variety during the EO extraction to obtain oils with desirable composition and aroma. However, current use of hemp extracts and claims are ahead of science; decades of prohibiting research and development on hemp created a substantial knowledge gap on hemp terpenes. Therefore, this research addressed the knowledge gap and the market needs. The hypothesis of this study was that one can modify and obtain EO chemical profile with desirable composition and antimicrobial activity from a single hemp cultivar. The objective of this study was to evaluate the effect of distillation time and grinding of hemp before EO extraction on EO profile and antimicrobial activity.

## 2. Results and Discussion

### 2.1. Essential oil (EO) Constituents and EO Yield

Eighty-nine (89) constituents were identified in the hemp EO, belonging to monoterpenes, sesquiterpenes and cannabinoids, although not all constituents were found in all of the distillation timeframe (DT) fractions (Supplementary Tables S2 and S3). The essential oil (EO) sampled at different time points during the hydrodistillation of either ground or non-ground hemp material had a dissimilar profile, as individual EO constituents come out at different times (Supplementary Tables S2 and S3). We statistically analyzed only 26 constituents, with the highest concentration in the EO. As indicated in the Materials and Methods section, Analysis of Variance (ANOVA) of a 2 × 5 factorial design was completed to determine the significance of the main and interaction effects of Material (two levels: Ground and Nonground) and Distillation Time (DT; 5 levels: 0–5 min, 5–10 min, 10–20 min, 20–80 min, and 80–120 min) on 26 constituents.

The ANOVA results shown in Table 1 revealed that only the main effects of Material and DT were significant on *α*-pinene, terpinolene, and cannabidiol (CBD); and the interaction effect of Material and DT was significant on all other constituents (EO content (yield), *β*-pinene, myrcene, *δ*-3-carene, limonene, eucalyptol, *β*-(*Z*)-ocimene, *β*-(*E*)-ocimene, *β*-caryophyllene, *α*-(*E*)-bergamotene, (*Z*)-*β*-farnesene, *α*-humulene, caryophyllenyl alcohol, germacrene d-4-ol, spathulenol, caryophyllene oxide, humulene epoxide 2, *β*-bisabolol, *α*-bisabolol, *δ*9-tetrahydrocannabivarin, cannabicyclol, cannabichromene, *δ*8-tetrahydrocannabinol, and *δ*9-tetrahydrocannabinol (dronabinol)). The corresponding multiple means comparison results are shown in the following tables.

Interestingly and despite the expectation that grinding of the material prior to EO would speed up the EO extraction, most of the EO in the non-ground material eluted early in the distillation process, while most of the EO in the ground material was eluted later, in the 20–80 min DT (Table 2). Grinding of the material has been shown to facilitate and to significantly speed up the extraction of EO in other crops such as anise [23] and junipers [24]. However, because hemp EO is synthesized and stored in trichomes, which are epidermal structures [25], volatile fractions can be extracted relatively fast, while the EO in junipers is synthesized and accumulated in endogenous EO structures. The observed delay in EO extraction from ground biomass may be due to different physical and chemical processes developing during the grinding of the material in water, then introducing the slurry in the Clevenger apparatus before extraction. This postulation merits further investigation.

Non-stop distillation of the hemp biomass for 180 min yielded 0.46% EO, while the EO yield of the ground material (sum of 0–120 min was 0.315%, and the EO yield of nonground material was 0.42%. These results demonstrated that the process of removing EO fractions did not compromise the EO yields. The EO content in this study was higher than that reported EO yield in cv. Fedora 17 [26] and also higher than the EO range of 0.11 to 0.25% (*w*/*w*) reported for 10 hemp cultivars [27]. Another recent study from Italy reported EO yields (of cv. CS (Carmagnola Selezionata) of 0.14 and 0.15% *w*/*w* for hydrodistillation and microwave-assisted distillations, respectively [28]. A recent study on industrial and wild hemp (*Cannabis sativa* spp. *spontanea* Vavilov) from the same location (as in this study) and using the same hydrodistillation method (without grinding) reported EO yields of 0.085–0.262 mL/100 g of air-dried material in wild hemp, and 0.06 to 0.14 mL/100 g in eight other registered industrial hemp cultivars [29]. These results may suggest that drying of hemp material may significantly reduce the EO yield.

### 2.2. Essential Oil Profile of Hemp EO Fractions as a Function of Distillation Time (DT) and Material

#### 2.2.1. *β*-Pinene

The concentration of *β*-pinene in the control EO was 3.1%, its concentration ranges were 0.75–7.0% and 3.7–8.3% in the EO fractions of the nonground and ground materials, respectively. *β*-Pinene was eluted early in the hydrodistillation of the ground material and decreased in later DT; however, the dynamics of its elution in the non-ground material was different, with maximum elution in the 80–120 and 0.5 DT and the lowest in the 20–80 min DT (Table 2). *β*-Pinene is a bicyclic monoterpene and along with the *α*-pinene, one of the determinants of hemp EO aroma. Its concentration in the control EO of this study was similar to previous reports [17,19,20,26,27,30]. The concentration of *β*-pinene was found to be dependent on location and harvest time and varied from 2.3 to 3.6% in the EO of cv. Fedora 17 [26], up to 9.2% in commercial hemp EOs [19], and was 2.2% in marijuana EO, [31].

#### 2.2.2. Myrcene

The concentration of myrcene in the control EO was 7.1%, similar to previous reports of 2.4–9.7% [19,30,32]. This constituent is one of the determinants of marijuana odor, has a high-odor impact, and can easily emit through duffel bag [33]. Myrcene concentration ranges in the EO fractions in this study were 3.1–15.5% and 12.5–21.1% in the fractions of the nonground and ground materials, respectively (Supplementary Table S1). Most of the myrcene in the ground material was eluted in the 5–10 min DT and much less in later DT (Table 2). However, myrcene in the EO from nonground material was the highest in the 80–120 min DT (Table 2). Myrcene, acyclic monoterpene, is one of the major constituents in hemp EO [17,20,27,32], either industrial (grain or fiber) or hemp used for production of high-value chemicals such as CBD in the U.S. [29]. Myrcene concentration in CBD-type hemp strain was reported to be 9.2–12% of total oil [29]. Myrcene in the EO of cv. CS (Carmagnola Selezionata) was 5–6% [17]. Myrcene in the drug-type hemp (marijuana) tissue was relatively low, (0.7–4.3%) in female flower buds [34], and it was 1% in the marijuana EO [31].

#### 2.2.3. *δ*-3-Carene

The concentration of *δ*-3-carene in the control EO was 4.5%, and its concentration ranges were 4.3–8.2% and 3.5–5.7% in the EO fractions from the nonground and ground materials, respectively (Table 2). The concentration of *δ*-3-carene was the highest in the 0–5 min DT from nonground material. *δ*-3-Carene is a bicyclic monoterpene, found in all types of hemp [35]. In a previous study, its concentration was found to be n.d.–62% in wild hemp EO, and n.d. to 0.19, 0.45, and 0.79% in the EO of hemp cultivars Carmagnola, Simba, and Dioica, respectively [29]. Recent studies reported n.d. to 0.1% *δ*-3-carene in the EO of cv. CS [17], and 0.2–0, and 0.2–0.3% in the EO of cv Felina 32 [28], and again 0.2% in the EO of Felina [36].

#### 2.2.4. Limonene

Limonene concentration in the control EO was 2.38%, which was within the range reported previously [22,26]. Limonene concentration in the EO of cv. Felina 32 was around 0.4–0.5% [28,36], and around 2% in the EO of cv. CS [17]. Limonene concentration ranges were n.d–4.11% and 1.8–2.8% in the EO fractions of the nonground and ground material, respectively (Supplementary Tables S2 and S3). The concentration of limonene was also the highest in the 0–5 min DT fraction of nonground material. In a previous study at the same location, limonene in the EO was n.d.–0.76% in feral hemps, and n.d.–0.29 in cv. Bacalmas, 0.59% in cv. Carmagnola, 0.92% in cv Helena, and 1.29% in cv Simba were [29]. However, the concentration of limonene in U.S. strain grown for commercial production of CBD was 6.5–7.5% [29]. Hemp “strain” is a common term in the U.S. to describe chemotype of hemp, or selection line that was not yet officially registered; there are hundreds of strains with names like cultivars, some are called “varieties” [37]. However, some authors argue it was inappropriate term to describe chemotypes, as this term is used for bacteria and viruses but not for plants [12]. Limonene, monocyclic monoterpene, is found in all types of hemp, it is one of the signature compounds in marijuana headspace [33]. For example, limonene concentration in drug-type hemp varied between 0.03 and 1.53% [34], the sum of limonene and *β*-phellandrene was reported to be 1% of marijuana EO [31].

#### 2.2.5. Eucalyptol (1,8-Cineol)

Eucalyptol (1,8-cineol) concentration in the control EO was 1.38%, and its ranges in the EO fractions were 0.00–2.65% and 0.2–4.3% in the nonground and ground materials, respectively. The concentration of eucalyptol was the highest in the 0–5 min ground and in the 80–120 min nonground material fractions. While the concentration of this constituent decreased in later DT fractions from the ground material, a somewhat reverse trend was observed with its concentration in the nonground material (Table 2). Eucalyptol, bicyclic monoterpenoid, is a common constituent of hemp EO [26,38], but also in the EO of other species such as lavender and hyssop [39], Scotch spearmint [40], common basil [41], and has shown antidepressant activity in rats [42]. The concentration of eucalyptol in the EO of hemp from the same region was reported to be n.d.–1.6% in nine wild hemps, and n.d.–2.4% in registered cultivars Carmagnola, Sequieni, Helena, Simba, Spic, and Bacalmas [29].

#### 2.2.6. *β*-(*Z*)-Ocimene and *β*-(*E*)-Ocimene Concentration

The concentration of *β*-(*Z*)-ocimene (acyclic monoterpene) in the control EO was 1.8%, and its ranges in the EO fractions were 0.00–2.7% and 0.96–4.1% in the nonground and ground materials, respectively (Supplementary Tables S2 and S3, Table 3). Its concentration was the highest in the 80–120 min DT fraction of the ground material (Table 3). Apparently, this is a genetic trait, as this constituent was not detected in the EO of nine other registered industrial hemp cultivars [29]. Interestingly, in a previous study, this constituent was found in most of the wild hemps in the region, ranging from n.d. to 0.174% of the oil [29].

The concentration of *β*-(*E*)-ocimene (another acyclic monoterpene) in the control EO was 8.85% and ranged in 2.3–15.3% and 9.98–17.6% in the ground and nonground materials, respectively (Supplementary Tables S2 and S3). Its highest concentration was in the 5–10 min DT fraction of the ground material. *β*-(*E*)-Ocimene has been reported by other authors [17,43]. Recently, this compound was also found in the wild hemp EO and ranged from n.d. to 1.63%, but it was not detected in the EO of 8 other registered industrial hemp cultivars [29].

#### 2.2.7. *β*-Caryophyllene

*β*-Caryophyllene concentration in the control EO was 9.1%, which was low compared with its concentration in the EO of other registered cultivars grown in the same location [29]. In the latter report, the concentration of *β*-caryophyllene was 15.4–26.8% in wild hemps, and 25–40% in eight registered hemp cultivars, and 6.5–7.5% in the EO of U.S. hemp strain grown for commercial production of CBD [29]. Apparently, different cultivars can accumulate and yield vastly different concentrations of *β*-caryophyllene even when they are grown in the same location and under the same agronomic practices. Overall, *β*-caryophyllene in the EO of this study was within previously reported ranges [20,27,43,44].

In this study, *β*-caryophyllene concentration ranges in the EO fractions were 8.3–37.7% and 5.4–12.8% in the nonground and ground materials, respectively (Supplementary Tables S2 and S3). Its highest concentration was found in the 80–120 min DT fraction of the nonground material (Supplementary Table S2, Table 3). Its concentration generally increased in later DT fractions (Table 3).

*β*-Caryophyllene, bicyclic sesquiterpene, is one of the major hemp EO constituents, be it from industrial hemp (grain and fiber) [38], US strains grown for non-psychoactive cannabinoids such as CBD and CBG [29], or in the drug-type (marijuana strains) [34,45]. This study demonstrated that while the concentration of *β*-caryophyllene was 8–10% in the control hemp EO; a 20% *β*-caryophyllene rich EO can be produced by collecting the EO in the 80–120 DT. This compound is a constituent of many other EOs and is known as a dietary cannabinoid; it can bind with high affinity to the CB2 cannabinoid receptor in peripheral tissues [44].

#### 2.2.8. *α*-(*E*)-Bergamotene

The concentration of *α*-(*E*)-bergamotene, a bicyclic sesquiterpene, was around 2.3% in the control EO, and 1.1–3.3% and 0.5–1.1% in the EO fractions of the nonground and ground materials, respectively. Its highest concentration was found in the 80–120 min DT fraction of the nonground material (Table 3). Interestingly, its concentration in the control oil was the highest, which might be an indication of converting some constituents in the collection part of the Clevenger apparatus during the hydrodistillation process. Generally, the concentrations of this EO constituent in the present study were comparable to the ones from a previous study at the same location but other cultivars, where the concentration range of *α*-(*E*)-bergamotene in the EO was 0.36–4.4% in the registered cultivars. The concentration of this constituent in the EO was similar to a previously reported concentration (3.2%) by Smerglio et al. [21], 4% by Zengin et al. [22], and 04–0.6% by Fiorini et al. [17].

#### 2.2.9. (*Z*)-*β*-Farnesene

The concentration of (*Z*)-*β*-farnesene, acyclic sesquiterpene, was around 2.35% in the control EO, 1.0–1.9% and 0.28–1.0% in the EO fractions from the nonground and ground materials, respectively (Supplementary Tables S2 and S3). The concentration of this constituent was the highest in the 80–120 min DT fraction of the nonground material. (*Z*)-*β*-farnesene, bicyclic sesquiterpene, was reported in the EO of hemps from the same region, with concentrations 0.4–3.0% in nine wild hemps, and n.d. to 3.3% of the EO of cv. Helena [29].

#### 2.2.10. *α*-Humulene

The concentration of *α*-humulene (*α*-caryophyllene), monocyclic sesquiterpene, was 4.4% in the control oil, 3.0–11.7% and 1.8–4.3% in the EO fractions, of the nonground and ground materials, respectively. The highest concentration of this constituent was found in the EO of the 80–120 min DT fraction of the nonground. This constituent has been reported in hemp EO by other researchers [17,26,43]. The EO of other hemps from the same region extracted using the same method for the same time duration had concentrations of 10.5–17.4 in eight registered hemp cultivars, 4.4–7.6% in eight new hemp lines, and 5.3–11.9% in eight wild hemps [29].

#### 2.2.11. Caryophyllenyl Alcohol

The concentration of caryophyllenyl alcohol, a bicyclic sesquiterpenoid, was 2.1% in the control EO, 0.88–3.17% and 0.54–1.5% in the EO fractions of the ground and nonground materials, respectively (Table 4; Supplementary Tables S2 and S3). Its highest concentration was in the 20–80 and 80–120 min DT fractions (Table 4). The concentrations of this constituent in the EO from other hemps in the same region were 0.17–1.19% in new hemp breeding lines [29].

#### 2.2.12. Germacrene D-4-ol

The concentration of the germacrene D-4-ol, a monocyclic sesquiterpenoid, was 1.63% in the control EO; its range was 0.71–3.45 in the EO fractions from the nonground material and 0.61–1.38 in the EO fractions from the ground material. Its highest concentration was found in the 80–120 min DT fraction (Table 4). Its overall concentration trend was towards an increase in the later DT fractions. This EO constituent was reported to vary from n.d. to 1.64% in eight registered hemp cultivars, 0.2–1.2% in eight new hemp breeding lines, and 0.1–1.9% in eight wild hemps [29]. Germacrene D varied from n.d. to 0.7% in 17 commercial hemp EOs [19].

#### 2.2.13. Spathulenol

The concentration of spathulenol, a tricyclic sesquiterpenoid, was 2.38% in the control EO, and its ranges in the nonground and ground materials were 0.58–2.88 and 0.79–2.35%, respectively. Its highest concentration was in the 80–120 min DT fraction of the nonground material. The trend was towards increasing its concentrations with increasing time. Generally, the concentration of this constituent was higher than in eight other registered hemp cultivars (n.d.–0.64%), and in most of wild hemps with the exception of one that had 2.5% spathulenol in its EO [29]. Spathulenol concentration in hemp EO was reported to be in 0.1–0.2% and was found in the EO of only few cultivars [19].

#### 2.2.14. Caryophyllene Oxide

The concentration of caryophyllene oxide, a bicyclic sesquiterpenoid, was 3.8% in the control EO, which was within the concentration ranges reported previously [17,19,21,22,26]. Its range in the EO fractions were 1.1–7.9% and n.d.–5.8% in the nonground and in ground materials, respectively. Its concentration was the highest in the 80–120 min EO fraction of nonground material. Overall, its concentration increased in later DT fractions of both ground and nonground materials. This study shows that if caryophyllene oxide is desirable in the EO, its concentration can be increased twofold by collecting EO eluted after 80 min. The concentration of caryophyllene oxide in the hemps EO from the same region was 8.6–16.6% in new hemp breeding lines, 4.2–6.6% in eight registered hemp cultivars, and 0.2–31% in eight wild hemps [29]. The concentration of caryophyllene oxide was reported to be 5.2–5.9% in the EO of cv. Carmagnola Selezionata [17], 0.5–9.5% in commercially available hemp EOs [19], and 7.4% in the EO of marijuana [31].

#### 2.2.15. Humulene Epoxide 2

The concentration of humulene epoxide 2 (bicyclic sesquiterpenoid) was 1.6% in the control EO, it ranged from 0.25 to 2.6% in the EO fractions of the nonground material and was 0.45–1.78% in the EO fractions of the ground material (Supplementary Tables S2 and S3). Overall, grinding decreased its concentration in the EO. There was a positive trend towards increased concentration of both ground and nonground materials with increasing DT (Table 4). The highest concentration was found in the 20–80 and 80–120 min DT EO fraction of nonground material (Table 4). Humulene epoxide 2 was previously reported in hemp EO from Italy [17]. Its concentrations in hemp EO from the same region were 1.3–9.5% in eight wild hemps, and 0.96–2.0% in eight registered hemp cultivars [29]. Humulene-1,2-epoxide was found in low concentrations; around 0.2% of 17 EOs and was absent in most of them [19], while it was 1.9–2.0% in the EO of cv. CS [17].

#### 2.2.16. *β*-Bisabolol

The concentration of *β*-bisabolol (monocyclic sesquiterpenoid) was 1.08% in the control EO, its concentrations in the EO fractions were n.d.–2.24% and n.d.–1.1% in the nonground and ground fractions, respectively (Supplementary Tables S2 and S3, Table 4). Overall, grinding decreased its concentration in the EO, and its concentrations increased in the later sampled fractions. Therefore, its highest concentration was in the 80–120 min DT fraction (Table 4). A monocyclic sesquiterpenoid, *β*-bisabolol, has been reported in other hemp EOs [19]. The concentration of *β*-bisabolol in hemp EO from the same region from a previous study was 0.89–1.99% in wild hemps, and from n.d. to 1.72 in eight registered hemp cultivars [29]. Other recent studies reported n.d. to 0.5% in the commercial hemp EOs [19].

#### 2.2.17. *α*-Bisabolol

The concentration of *α*-bisabolol, monocyclic sesquiterpenoid, was 1.33% in the control EO, its concentrations in the EO fractions were n.d.–2.63 in the nonground material and n.d.–1.68 in the ground fractions (Supplementary Tables S2 and S3, Table 5). Generally, grinding reduced the amount of *α*-bisabolol in the EO, and its concentration increased with in later sampling times. Therefore, its concentration was the highest in the 80–120 min DT fraction of the nonground material (Table 5). *α*-Bisabolol was reported in commercial hemp EO [17]. The concentration of *α*-bisabolol in hemp EO from the same region were 0.1–2.8% in wild hemps, and from n.d. to 6.7% in eight other registered hemp cultivars [29]. The concentration of *α*-bisabolol in the EO of cv. Carmagnola Selezionata was 0.4–0.5% of the relative peak area [17], and below 1% in 17 commercial EOs [19].

#### 2.2.18. Monoterpenes

As expected, most of the monoterpenes were eluted early in the distillation process and therefore, their concentrations were the highest in the initial DT fraction, and gradually decreased in the later fractions (Table 5). The concentration of the monoterpenes in the control EO was 44.4%, which was within the concentration range of monoterpenes (31% and 83% of the total peak area) identified in 17 commercial hemp EO [19] and their concentration (around 41%) in hemp cv. Futura 75 EO [21].

The monoterpenes concentration in the EO fractions was 12.9–76.9% and 54.5–83.9% in the nonground and ground materials, respectively. Grinding increased the amount of monoterpenes in the EO. The amount of monoterpenes was 44.9% in the control EO. The highest concentration of monoterpenes was found in the 0–5 and 5–10 min DT fractions of the ground material. The results showed that the total concentration of monoterpenes in hemp EO could be significantly modified by grinding and distillation time. The total amount of monoterpenes in the EO of other hemps from the same region was reported to be from n.d. in two wild hemps to 8.0% in other wild hemps, and 1.4–13.5% in eight registered hemp cultivars [29]. Total monoterpenes in nine drug-type varieties of hemp were 0.5–8.4% in dry flowers [34], however, this cannot be readily related to their potential concentration in the EO.

#### 2.2.19. Sesquiterpenes

Converse trends were observed with the amount of sesquiterpenes. The amount of sesquitepenes was 46.9% in the control EO, and their concentration ranges in the EO fractions were 21.1–80.3% and 13.7–42.3% in the nonground and ground materials, respectively. Grinding decreased the amount of sesquiterpenes in the EO fractions, while later sampling times increased it. Therefore, the highest amount of sesquiterpenes was found in the 80–120 min DT of nonground material (Table 5). The amount of total sesquiterpenes in hemp EO from the same region was 69.7–89.1% in 8 new hemp breeding lines, and 75.5–79.2% in eight other registered hemp cultivars [29]. Sesquiterpenes were reported to be around 57% in the EO of cv. Futura 75 [21]. Another recent study found the sesquiterpenes and monoterpenes had similar concentration in two out of 17 commercial hemp oils, and sesquiterpenes were higher than monoterpenes in additional two of the 17 hemp EOs [19]. Greater concentration of sesquiterpenes in hemp EO is an indication of aging [19]. In another study, sesquiterpenes were between 29 and 48% of the EO depending on the pretreatment and extraction methods [28].

#### 2.2.20. *α*-Pinene and Terpinolene

The concentrations of *α*-pinene and terpinolene were greater in the EO fractions from the ground material compared with the ones in the nonground material (Table 6). The concentrations of both constituents were much higher in the initial DT EO fractions compared with the control EO. This was expected because the two constituents are known to be some of the most volatile ones in hemp EO [28]. While the concentration of *α*-pinene in the control EO was 3.2%, grinding of the material resulted in 14.8% of *α*-pinene overall in all fractions. *α*-Pinene was identified as one of the determinants of hemp scent emitting by the plant [43]. The concentration of *α*-pinene in this study (14.9% of the total oil) was similar to its concentrations reported in the literature: 11% [22], 17.0% [36], and 11–17% [20]. In a recent study on registered cultivars in the same region, *α*-pinene in the EO varied from n.d. to 8.4% [29], indicating that genetics plays a key role in the accumulation of this bicyclic monoterpene in hemp. The results from this study on *α*-pinene are similar to previous reports following either steam or hydrodistillation [27], although a bit lower concentration of *α*-pinene have been reported [20,36]. Total sesquiterpenes in nine drug-type hemp varieties was 0.16–0.93% in dried flowers [34].

#### 2.2.21. Cannabinoids

The sum of the cannabinoids identified in this study (*δ*9-tetrahydrocannabivarin, cannabicyclol, cannabidiol (CBD), cannabichromene, *δ*8-tetrahydrocannabinol, *δ*9-tetrahydrocannabinol (dronabinol)) was 6.58% in the control EO; cannabinoids concentration in the EO fractions were 0–5.97% and 0–6.12% from the nonground and ground material, respectively. These results suggest a likelihood for some type of conversion happening during the 180 min non-stop distillation that results in higher cannabinoids content in the EO. It has been known that THC and CBD are not formed enzymatically in hemp; they are decarboxylated forms of CBDA and THCA, and postharvest processing plays a significant role in their formation [12]. In this study, cannabinoids were eluted late in the distillation process, consequently resulting in larger amount in the later collected EO fractions of both ground and nonground materials. The highest concentration of cannabinoids was found in the 80–120 min DT fraction of the ground material (Table 5). The amount of total cannabinoids in the EO of hemps from the same region were 9.9–20.7% in eight registered hemp cultivars [29]. Total cannabinoids in a study of 17 industrial hemp EOs was low [19]. The amount of total cannabinoids in nine drug-type varieties of hemp ranged between 12.2 and 28.4% on dry matter in flowers [34]. However, these results were in dry matter (dried hemp flowers) and not in EO.

#### 2.2.22. Cannabidiol (CBD) Concentration in EO Fractions

Grinding resulted in a marginally significant decrease in the CBD concentration of the EO (Table 6). Most of the CBD came out later in the distillation process; therefore, its highest concentration (5–6%) was in the 120–180 min DT fraction. The concentration of CBD in the control EO was 5.67%, while its concentration in the EO fractions was n.d.–5.27% and n.d.–6.124 in the nonground and ground materials, respectively. In a recent study on the EO from wild hemps and registered cultivars, the concentration of CBD in the EO of wild hemp was 6.9–52.4%, and CBD in the EO of the registered cultivars was 7.1–25.4%, and its content in one CBD strain from U.S. varied from 7.4 to 8.8% [29]. The concentration of CBD in 24 cultivars of industrial hemp varied from 0.58 to 1.83% of dried biomass [46]. Cannabidiol concentration in 17 commercial EOs was low, below 0.4% of the total oil [19].

Cannabinoids are of significant interest recently, especially non-psychoactive phytocannabinoids such as CBD, as the market expanded dramatically [11]. Recently, there has been a flash of research articles on CBD effects on human health [9,11,47]. Previous research has shown that CBD has anti-epileptic, anti-inflammatory, and anti-psychotic properties [48] and against neurodegenerative diseases [9]. A recent review summarized the various molecular pathways that cause positive effects of CBD, especially with respect to the major neurodegenerative disorders [9]. The author elaborated on published data that support the notion of CBD as a potential pharmacological tool for the treatment of such disorders, and CBD safety as a potential therapeutic agent [9].

#### 2.2.23. THC (*δ*9-Tetrahydrocannabinol) Concentration in the EO Fractions

The concentration of THC (*δ*9-tetrahydrocannabinol or Dronabinol) was 0.142% in the control EO, and its ranges were n.d.–0.11% and n.d.–0.1% in the fractions from the nonground and ground material, respectively (Supplementary Tables S2 and S3). This study showed that THC-free EO can be obtained if the EO distillation is limited to 120 min. However, this will also reduce the amount of CBD and sesquiterpenes in the EO. The concentration of THC is regulated internationally, e.g., most countries require that the THC concentration in hemp be below 0.3% and in some countries such as France it must be below 0.2% dry weight (Regulation EC No. 1124/2008, Annex XII). In the U.S., hemp with THC > 0.3% on dry weight basis is considered marijuana. Registered industrial hemp cultivars (varieties) differ in their THC concentration, but generally, it is very low. In a study of 24 industrial hemp cultivars, THC varied from 0.03 (in cv. USO-13) to 0.75% (in cv. Secuieni 1) in dry matter [46]. The THC concentration in the EO of hemps from the same region was from n.d. to 3.4% in wild hemps, and from n.d. in the EO of cvs. Bacalmas, CS, Spic, Dioica, and Helena, to 0.42% in Sequieni, 0.76% in cv. Simba, and 0.96% in cv. Carmagnola [29]. The THCA concentration in drug-type hemp may be very high, e.g., THCA was 3.2–26.3% dry weight of flowers from varieties Blackberry Kush, Black Lime, Canna Tsu, Cherry Chem, Valley Fire, Mamma Thai, Sour Diesel, Terple, and White Cookies [34]. However, the CBD and CBDA concentrations in the above varieties were very low, mostly below 1% with the exception of Canna Tsu [34].

#### 2.2.24. Concluding Discussion on Terpenes and Cannabinoids Concentration in the EO Fractions

Hemp control EO and the hemp EO fractions in this study had differential chemical composition, they both are mixture of terpenes and cannabinoids, probably non-psychotropic due to the very low concentrations of THC; however they may possess various types of bioactivity including in humans, due to “entourage-effect” as described previously [2,11,12]. This study demonstrated that EO fractions can be obtained from a single hemp cultivar by modulating grinding before distillation and fractions generation in time. Indeed, this study provided hemp EOs with greater diversity of mixture of terpenes and cannabinoids compared with the 17 commercially available hemp EO from a number of hemp cultivars: Antàl (EO1), Bielobrzerski (EO2), Carmagnola (EO3), Carmagnola CS (EO4), Dioica 88 (EO5), Fedora 17 (EO6), Ferimon (EO7), Finola (EO8), Futura 75(EO9), KC Virtus (EO10), KC Zuzana (EO11), Markant (EO12), Santhica 27 (EO13), Santhica 70 (EO14), Tiborszallasi (EO15), Tygra (EO1) and Zenith (EO17) [19].

#### 2.2.25. Antimicrobial Activity of the Hemp EO Fractions

In this study, standard antibiotics cefoxitin for Gram-negative (G^−^) bacteria and gentamicin for Gram-positive (G^+^) bacteria, and fluconazole for yeast were used as positive control: The G^−^ microorganisms included: SE—*Salmonella enterica* subsp. *enterica*, PA—*Pseudomonas aeroginosa*, YE—*Yersinia eneterocolitica*. The G^+^ microorganisms included in this study were SA—*Staphylococcus* subsp. *aureus*, EF—*Enterococcus faecalis*, SP—*Streptococcus pneumonia*. Finally, the yeast used in this study included CA—*Candida albicans*, CK—*Candida krusei*, and CT—*Candida tropicalis*.

The Analysis of Variance (ANOVA) results that show the significance of the main and interaction effects of Material (2 levels: Ground and Nonground) and Distillation time (DT; 6 levels: 0–5 min, 5–10 min, 10–20 min, 20–80 min, 80–120 min, and 120–160 min) on nine these antimicrobial activities (SA, EF, SP, PA, YE, SE, CA, CK, CT) are presented in Table 7. The results indicate that the interaction effect was not significant on SA and SE, but the main effect of Material was significant on SA, and the main effect DT was significant on SA and SE. The interaction effect of Material and DT was significant on EF, PA, SP, YE, CA, CK, and CT (Table 7).

Comparison of the means showed that the 20–80 min DT EO fraction from ground material had the highest antimicrobial activity against EF, SP, and PA (Table 8). The highest activity against YE was demonstrated at the 20–80 min EO fraction from the nonground material, the highest activity against CA and CK was found in the 0–5 min EO fraction from the nonground material, and the highest activity against CT was found with the use of the 5–10 min DT EO fraction from ground material (Table 8).

As positive control, the antibiotics gentamycin for G^+^, cefoxitin for G^−^ and fluconazole for yeasts were used. Inhibition zones of the antibiotics were generally greater in comparison with inhibition zones of the essential oil fractions. In this study, the antibiotics had greater effect on bacteria than on yeasts. The absence of outer cell membrane makes the bacteria vulnerable to the effect of antibiotics. This argument is supported by previous reports on G^+^ and G^−^ bacteria and their resistance to antibiotics [49,50]. The outer cell membrane present in G^−^ bacteria is thought to play an important role as a protective mechanism against antimicrobial agents and antibiotic selection pressure [51].

Overall, the EO fractions from the ground material had higher antimicrobial activity against the tested microorganisms compared to those from nonground materials (Table 9). The highest activity against SA was recorded by the use of either the 5–10 min or 20–80 min DT EO fraction and the highest activity against SE was recorded by the use of the 10–20 min DT EO fraction (from either ground or nonground materials) (Table 9).

The antimicrobial activity results may be due to significant differences in EO composition between the different EO fractions. The effects may be a direct result of a single constituent, although in most cases the observed bioactivity effect has been shown to be a result of the synergistic effects of 2 or more constituents [52].

Hemp extract using organic solvent exhibited very good antimicrobial activity against *S. aureus* [53]. The hemp leaves extracts fraction exhibited activity both against G^+^ and G^−^ bacteria and against the fungi. However, the aqueous extract did not show any antimicrobial activity [54]. Researchers have reported antibacterial activity of cannabinoids against a wide range of bacteria [55,56,57]. The aqueous and alcoholic extracts of hemp seeds did not show any antibacterial activity against *P. mirabilis* [58]. In the results of Nissen et al. [20] promising inhibitory activities of hemp oils against G^+^ opportunistic/pathogens such as *Clostridium* spp. and *Enterococcus* spp. was shown. The results of Iseppi et al. [19] showed a good antibacterial activity of six hemp EOs against the G^+^ bacteria, thus suggesting that hemp EO can inhibit or reduce bacterial proliferation and it can be a valid support to reduce microorganism contamination, especially in the food processing field.

## 3. Materials and Methods

### 3.1. Plant Material, Growing Conditions, and Sampling

Certified seeds of industrial hemp (*Cannabis sativa* L.) cv. Novosadksa were provided by the Institute for Field and Vegetable Crops in Novi Sad, Serbia. Cultivar Novosadksa is a dioecious hemp, included in the European List of Approved hemp varieties [59]. “Novosadska konoplja” is an improved selection from “Flajsmanova”, which is the same as Italian origin “Fleischmann hemp” [46]. It was selected in the 1950s but included in the former Yugoslav cultivar register only since 1989. “Novosadska konoplja” is presently maintained and commercialized by the Institute of Field and Vegetable Crops, Novi Sad, Serbia.

Field trials were established in Backi Petrovac, Serbia (45.336500° N 19.671355° E), at the Alternative Crops and Organic Production Department, a research unit of the Field and Vegetable Crops in Novi Sad, Serbia. Cultivar Novosadska along with other cultivars was grown in a randomized complete block design in three replicates, with a size of the individual plots 2 m by 5 m.

The soil at the Research Station at Backi Petrovac is alluvial chernozem with pH of 7.2. Fertilization was performed following the guidelines for fiber hemp and included broadcast application of NPK 16:16:16 at 300 kg/ha prior to plowing in the fall. The soil was prepared after the harvest of millet, the previous crop. Soil preparation consisted of deep plowing in 12 November 2018, followed by disking and cultivation in 25 March 2019 for the establishment of a suitable seedbed. The traditional industrial hemp (grain and/or fiber) in most of northern, middle and southeastern Europe is grown as rainfed, without irrigation; it is traditionally planted early (March–April) and because of its deep fast-growing root system it is able to efficiently utilize winter moisture accumulated in the soil. Therefore, hemp cv. Novosadksa was also grown without irrigation. The crop was seeded on 27 March 2019 at between row spacing of 50 cm and seeding rate of 30 kg/ha with a corn planter as a row crop. During the vegetation, plots were kept weed-free by mechanical cultivation between the rows during the initial vegetative stages until the crop closes the canopy (around 5 weeks after emergence). Hemp was left to form seed and was sampled for essential oil (EO) extraction in 2 October 2019 by cutting the top 50 cm of female plants from three plots.

### 3.2. Essential Oil (EO) Extraction Experiments

The EO extraction was conducted via hydrodistillation in three replicates of fresh hemp biomass in 4-L hydrodistillation Clevenger-type units. Each sample was 300 g of fresh biomass in 1.5 L water, as described previously for hemp EO extraction [29]. Each sample of 300 g of fresh material, included leaves flowers, seeds and seed bracts; all stems were removed.

Two experiments were conducted using the same biomass samples so the results can be comparable: (1) with nonground material, and (2) with ground material. Grinding of the samples for the second experiment was done using kitchen mixer for 30 s in water to avoid EO loss.

Two replicates were included in this report because that was sufficient for the statistical analyses. Beginning of the distillation was noted when the first drop of EO was eluted out of the condenser and deposited in the collection part of the Clevenger apparatus. All samples were distilled non-stop for 180 min. At the end of the distillation, the heat source was removed, the EO was measured in the graduated part of the apparatus by volume, the EOs (along with some water) were collected in glass vials and placed in a freezer. After all distillations were completed, the EO was separated from water, measured on an analytical scale, and kept in a freezer until the gas chromatography (GC) analyses could be performed. Herewith, we report the EO as volume by weight in hemp fresh material.

### 3.3. GC Analyses of Hemp Essential Oils (EOs)

The composition of hemp EOs was characterized by 7890A gas chromatograph (Agilent Technologies Inc., Santa Clara, CA, USA) coupled to 5975C mass selective detector (MSD). HP-5ms silica fused capillary column (30 m length × 0.32 mm i.d. × 0.25 µm film thickness) was used for this purpose.

The oven temperature program used was: 40 °C for 0min, increased to 300 °C with 5 °C/min, held for 10 min. The flow rate of the carrier gas (He) was 1.0 mL/min. The injection volume was 1.0 µL at split ratio 20:1. The temperatures of the ionization source, the quadrupole and the injector were 230 °C, 150 °C, and 250 °C respectively. The MSD was operated in full scan mode and all mass spectra were obtained at 70 eV in EI mode. The constituents present in the EO samples were identified by comparing their linear retention indices (LRI) and MS fragmentation patterns with those from the NIST′08 and Adams mass spectra libraries. The estimated LRI were determined using a mixture of aliphatic hydrocarbons (C8 to C40) under the same conditions described above.

The GC-FID analysis of the EOs was conducted with a 7890A gas chromatograph (Agilent Technologies Inc., Santa Clara, CA, USA) coupled to a FID and HP-5 silica fused capillary column (30 m length × 0.32 mm i.d. 0.25 µm film thickness). The oven temperature was programmed as mentioned above, whereas the detector and injector temperatures were 280 °C and 220 °C, respectively. The carrier gas was helium at a flow rate of 1.0  mL/min. EOs (1.0  μL) were injected using the split mode. The percentage composition of EO samples was calculated using the peak normalization method.

### 3.4. Antimicrobial Assay of Hemp Essential Oils (EOs)

#### 3.4.1. Bacteria and Yeasts Culture

The microorganisms used for antimicrobial activity testing in this study included three G^−^ bacteria *Pseudomonas aeroginosa* CCM 1959 (PA), *Salmonella enterica* subsp. *enterica* CCM 3807 (SE), *Yersinia enterocolitica* CCM 5671 (YE), three G^+^ bacteria *Enterococcus faecalis* CCM 4224 (EF), *Staphylococcus aureus* subs. *aureus* CCM 4223 (SA), *Streptococcus pneumonia* CCM 4501 (SP) and three yeasts *Candida albicans* CCM 8186 (CA), *C. krusei* CCM 8271 (CK), and *C. tropicalis* CCM 8223 (CT) (Czech Collection of Microorganisms, Brno, Czech Republic). The bacteria cultures were incubated in Mueller Hinton broth (MHB, Oxoid, Basingstoke, UK) at 37 °C, and yeast cultures were in Sabouraud broth (SB, Oxoid, Basingstoke, UK) at 25 °C overnight.

#### 3.4.2. Disc Diffusion Method

For the agar disc diffusion method, a 100 µL of 106 CFU/mL bacterial suspensions after incubation was spread on the Mueller Hinton Agar (MHA, Oxoid, Basingstoke, UK). Filter paper discs (6 mm in diameter) were infused with 15 µl of the EO tested and placed on the inoculated MHA. MHA was kept at 4 °C for 2 h and then at 37 °C for 24 h. For yeasts, 100 µL 10^6^ CFU/mL the yeast suspension was spread on Sabouraud agar (SA, Oxoid, Basingstoke, UK) and agars were cultivated at 25 °C for 24 h. After the incubation period, the diameter of inhibition zones was measured (mm). Growth inhibition was compared with the standard drugs. The standard drugs cefoxitin for G^−^ bacteria, gentamycin for G^+^ bacteria and fluconazole for yeasts were used as positive controls. Tests were performed in three separate experiments, and the means were calculated.

### 3.5. Statistical Methods and Analyses

For the gas chromatography results, Analysis of Variance (ANOVA) of a 2 × 5 factorial design was completed to determine the significance of the main and interaction effects of Material (2 levels: Ground and Nonground) and Distillation time (DT; 5 levels: 0–5 min, 5–10 min, 10–20 min, 20–80 min, and 80–120 min) on Essential oil (EO) and 26 constituents (*α*-pinene, *β*-pinene, myrcene, *δ*-3-carene, limonene, eucalyptol, *β*-(*Z*)-ocimene, *β*-(*E*)-ocimene, terpinolene, *β*-caryophyllene, *α*-(*E*)-bergamotene, (*Z*)-*β*-farnesene, *α*-humulene (*α*-caryophyllene), caryophyllenyl alcohol, germacrene D-4-ol, spathulenol, caryophyllene oxide, humulene epoxide 2, *β*-bisabolol, *α*-bisabolol, *δ*9-tetrahydrocannabivarin, cannabicyclol, CBD, cannabichromene, *δ*8-tetrahydrocannabinol, and *δ*9-tetrahydrocannabinol (dronabinol)), and 4 groups (monoterpenes, sesquiterpenes, cannabinoids, and Others(acid esters, ketone, alkochol) from ground and non-ground materials.

For the antimicrobial activity results, ANOVA of a 2 × 6 factorial design, with the two factors being Material (two levels: Ground and Nonground) and Distillation time (DT; 6 levels: 0–5 min, 5–10 min, 10–20 min, 20–80 min, 80–120 min, and 120–160 min), was completed to determine the significance of the effects on 9 antimicrobial activities (SA, EF, SP, PA, YE, SE, CA, CK, and CT).

The analyses were completed using the Mixed Procedure of SAS [60]. For significant (*p* < 0.05) effects, multiple means comparison was completed using Tukey’s multiple range test at 5% level of significance and letter groupings were generated. For each response variable, the validity of model assumptions was verified by examining the residuals as described in Montgomery [61].

## 4. Conclusions

The results from this study confirmed the hypothesis; this study generated hemp EOs with greater diversity of mixture of terepenes and cannabinoids compared with the 17 commercially available hemp EO from a number of hemp cultivars. The highest concentrations of *β*-pinene and myrcene in the EO can be obtained in the 5–10 min DT of ground material or in the 80–120 min DT of nonground material. If high concentrations of *δ*-3-carene and limonene are desirable, the EO can be obtained from 0–5 min DT fraction and the material is nonground. If higher concentration of the eucalyptol is desired, the EO can be sampled either in the 0–5 min DT of the ground material or in the 80–120 min nonground material. Overall, the highest concentrations of *β*-caryophyllene, *α*-(*E*)-bergamotene, (*Z*)-*β*-farnesene, *α*-humulene, (*α*-caryophyllene), caryophyllenyl alcohol, germacrene D-4-ol, spathulenol, caryophyllene oxide, humulene epoxide 2, *β*-bisabolol, *α*-bisabolol, sesquiterpenes, and CBD can be obtained when EO is sampled in the 80–120 min DT and the material is nonground. Fractions of hemp EO with high concentration of *β*-caryophyllene can be obtained by collecting the EO in the 80–120 DT. Monoterpenes in the hemp EO can be increased almost twofold to 85% by grinding the material before distillation and collecting the EO in the first 10 min. This is almost twice as high as their amount in the control EO (44.4%). Grinding increased the amount of monoterpenes in the EO. Sesquiterpenes can be increased up to 80% in hemp EO if the material if nonground and the EO is collected after 80 min DT. Sesquiterpenes concentration in the control EO were 46.9%. Grinding decreased the amount of sesquiterpenes in the EO fractions, while later sampling times increased it. Also, the concentration of *α*-pinene can be significantly increased by grinding of the material in water prior to distillation. Grinding resulted in slight be significant decrease in the CBD concentration of the EO (Table 6). Most of the CBD came out later in the distillation process; therefore, its highest concentration (5–6%) was in the 120–180 min DT fraction.

This study showed that THC-free EO can be obtained if the EO distillation is limited to 120 min. However, this will also reduce the amount of CBD and sesquiterpenes in the EO.

The best antimicrobial activity of hemp EO was against *Staphylococcus aureus* subsp. *aureus* and *Salmonella enterica* subsp. *enterica*, whole EO and EO fractions against two G^+^, two G^−^ and all *Candida* species, EO fraction from the nonground was found against *Yersinia eneterocolitica* and EO fraction from nonground materials against *S. aureus* subsp*. aureus*.

## Figures and Tables

**Table 1:** ANOVA *p*-values (*p*) that show the significance of the main and interaction effects of Material and Distillation Time (DT) on 26 response variables. *P*-values of significant effects that require multiple means comparison are shown in bold.

Source of Variation	Material	DT	Material*DT
EO	0.127	0.001	**0.001**
*α*-pinene	**0.001**	**0.001**	0.098
*β*-pinene	0.001	0.001	**0.001**
myrcene	0.001	0.001	**0.001**
*δ*-3-carene	0.001	0.001	**0.001**
limonene	0.323	0.001	**0.001**
eucalyptol	0.001	0.001	**0.001**
*β*-(*Z*)-ocimene	0.001	0.001	**0.001**
*β*-(*E*)-ocimene	0.001	0.001	**0.001**
terpinolene	**0.001**	**0.001**	0.101
*β*-caryophyllene	0.001	0.001	**0.001**
*α*-(*E*)-bergamotene	0.001	0.001	**0.001**
(*Z*)-*β*-farnesene	0.001	0.001	**0.024**
*α*-humulene (*α*-caryophyllene)	0.001	0.001	**0.001**
caryophyllenyl alcohol	0.001	0.001	**0.002**
germacrene D-4-ol	0.001	0.001	**0.001**
spathulenol	0.001	0.001	**0.001**
caryophyllene oxide	0.001	0.001	**0.001**
humulene epoxide 2	0.001	0.001	**0.001**
*β*-bisabolol	0.001	0.001	**0.001**
*α*-bisabolol	0.001	0.001	**0.005**
CBD	**0.057**	**0.001**	0.162
Monoterpenes	0.001	0.001	**0.001**
Sesquiterpenes	0.001	0.001	**0.001**
Cannabinoids	0.064	0.001	**0.001**
Others (acid esters, ketone, alkohols)	0.001	0.001	**0.001**

**Table 2:** Mean Essential oil (EO) content (*v*/*w* fresh material) and concentration (% of total oil) of *β*-pinene, myrcene, *δ*-3-carene, limonene, and eucalyptol obtained from the 10 combinations of Material and Distillation time (DT).

Material	DT (min)	EO (*v*/*w*)	*β*-Pinene	Myrcene	*δ*-3-Carene	Limonene	Eucalyptol
Ground	0–5	0.075 ^bcd^	7.56 ^ab^	18.8 ^b^	5.66 ^bc^	3.04 ^bc^	5.65 ^a^
Ground	5–10	0.030 ^de^	8.06 ^a^	20.7 ^a^	5.32 ^bcd^	2.47 ^de^	4.19 ^b^
Ground	10–20	0.030 ^de^	6.92 ^bc^	18.4 ^b^	5.09 ^cd^	2.68 ^bcd^	2.51 ^c^
Ground	20–80	0.095 ^b^	5.67 ^de^	13.9 ^cd^	4.58 ^cde^	2.66 ^cd^	0.84 ^d^
Ground	80–120	0.085 ^bc^	3.84 ^f^	12.7 ^d^	3.54 ^ef^	1.85 ^f^	0.23 ^e^
Nonground	0–5	0.200 ^a^	6.86 ^bc^	15.3 ^c^	8.17 ^a^	4.03 ^a^	2.59 ^c^
Nonground	5–10	0.050 ^bcde^	6.16 ^cd^	14.3 ^c^	6.26 ^b^	3.22 ^b^	1.05 ^d^
Nonground	10–20	0.040 ^cde^	5.16 ^e^	14.2 ^cd^	5.66 ^bc^	2.80 ^bcd^	nd
Nonground	20–80	0.055 ^bcde^	3.26 ^f^	10.7 ^e^	4.31 ^de^	1.94 ^ef^	nd
Nonground	80–120	0.075 ^bcd^	7.56 ^ab^	18.8 ^b^	5.66 ^bc^	3.04 ^bc^	5.65 ^a^

Within each column: means followed by the same letter are not significantly different at *α* = 5%. nd = not detected.

**Table 3:** Mean concentration (%) of *β*-(*Z*)-ocimene, *β*-(*E*)-ocimene, *β*-caryophyllene, *α*-(*E*)-bergamotene, (*Z*)-*β*-farnesene, and *α*-humulene (*α*-caryophyllene) obtained from the 10 combinations of Material and Distillation time (DT).

Material	DT (min)	*β*-(*Z*)-Ocimene	*β*-(*E*)-Ocimene	*β*-Caryophyllene	*α*-(*E*)-Bergamotene	(*Z*)-*β*-Farnesene	*α*-Humulene (*α*-Caryophyllene)
Ground	0–5	2.186 ^c^	15.98 ^ab^	6.31 ^ef^	0.684 ^e^	0.567 ^ef^	2.25 ^fg^
Ground	5–10	2.053 ^c^	17.30 ^a^	5.65 ^f^	0.510 ^f^	0.289 ^g^	1.93 ^g^
Ground	10–20	2.121 ^c^	15.94 ^ab^	6.33 ^ef^	0.513 ^f^	0.428 ^fg^	2.32 ^fg^
Ground	20–80	3.938 ^a^	13.19 ^c^	7.30 ^de^	0.710 ^e^	0.695 ^e^	2.95 ^efg^
Ground	80–120	1.248 ^e^	12.45 ^c^	10.57 ^c^	1.117 ^d^	1.014 ^d^	4.17 ^cd^
Nonground	0–5	2.683 ^b^	15.09 ^b^	8.64 ^d^	1.321 ^c^	1.190 ^c^	3.13 ^def^
Nonground	5–10	1.774 ^cd^	14.95 ^b^	11.73 ^c^	1.143 ^d^	1.101 ^cd^	4.03 ^cde^
Nonground	10–20	1.480 ^de^	13.07 ^c^	12.12 ^c^	1.075 ^d^	1.052 ^cd^	4.78 ^c^
Nonground	20–80	1.384 ^de^	9.91 ^d^	13.90 ^b^	1.494 ^b^	1.421 ^b^	5.87 ^b^
Nonground	80–120	0.659 ^f^	6.88 ^e^	20.64 ^a^	2.037 ^a^	1.745 ^a^	7.40 ^a^

Within each column, means followed by the same letter are not significantly different at *α* = 5%.

**Table 4:** Mean caryophyllenyl alcohol, germacrene D-4-ol, Spathulenol, caryophyllene oxide, humulene epoxide 2, and *β*-bisabolol obtained from the 10 combinations of Material and Distillation time (DT).

Material	DT (min)	Caryophyllenyl Alcohol	Germacrene D-4-ol	Spathulenol	Caryophyllene Oxide	Humulene epoxide 2	*β*-Bisabolol
Ground	0–5	0.55 ^f^	0.56 ^d^	0.64 ^e^	1.37 ^f^	0.25 ^f^	nd
Ground	5–10	0.55 ^f^	0.62 ^d^	0.81 ^de^	nd	0.46 ^ef^	nd
Ground	10–20	0.64 ^ef^	0.68 ^d^	0.96 ^de^	2.82 ^e^	0.75 ^de^	0.09 ^f^
Ground	20–80	1.04 ^cd^	0.94 ^c^	1.66 ^c^	4.23 ^d^	1.26 ^bc^	0.54 ^d^
Ground	80–120	1.49 ^b^	1.35 ^b^	2.24 ^b^	5.68 ^bc^	1.64 ^b^	0.83 ^c^
Nonground	0–5	0.86 ^de^	0.72 ^d^	0.59 ^e^	1.08 ^f^	0.25 ^f^	nd
Nonground	5–10	1.17 ^c^	1.00 ^c^	1.17 ^d^	3.49 ^de^	0.95 ^cd^	0.22 ^e^
Nonground	10–20	1.28 ^bc^	1.07 ^c^	1.66 ^c^	5.36 ^c^	1.50 ^b^	0.47 ^d^
Nonground	20–80	1.97 ^a^	1.51 ^b^	2.50 ^ab^	6.36 ^b^	2.24 ^a^	1.04 ^b^
Nonground	80–120	2.19 ^a^	2.08 ^a^	2.66 ^a^	7.66 ^a^	2.36 ^a^	1.40 ^a^

Within each column, means followed by the same letter are not significantly different at *α* = 5%. nd = not detected.

**Table 5:** Mean *α*-bisabolol, monoterpenes, sesquiterpenes, cannabinoids, and others (acid esters, ketone, alkochol) obtained from the 10 combinations of Material and Distillation time (DT).

Material	DT (min)	*α*-Bisabolol	Monoterpenes	Sesquiterpenes	Cannabinoids	Others (Acid Esters, Ketone, Alcohols)
Ground	0–5	nd	83.6 ^a^	14.2 ^g^	nd	0.57 ^a^
Ground	5–10	nd	84.8 ^a^	13.2 ^g^	nd	0.51 ^b^
Ground	10–20	0.48 ^e^	78.7 ^b^	19.6 ^f^	nd	0.45 ^c^
Ground	20–80	1.23 ^c^	66.7 ^c^	30.1 ^e^	1.74 ^c^	0.23 ^d^
Ground	80–120	1.65 ^b^	52.1 ^e^	41.9 ^c^	4.23 ^a^	0.41 ^c^
Nonground	0–5	nd	76.8 ^b^	21.3 ^f^	nd	0.56 ^a^
Nonground	5–10	0.33 ^e^	67.9 ^c^	30.1 ^e^	nd	0.21 ^d^
Nonground	10–20	0.85 ^d^	60.1 ^d^	37.9 ^d^	0.33 ^d^	0.16 ^e^
Nonground	20–80	1.67 ^b^	45.5 ^f^	51.2 ^b^	1.76 ^c^	0.11 ^f^
Nonground	80–120	2.18 ^a^	30.5 ^g^	64.8 ^a^	3.45 ^b^	nd

Within each column, means followed by the same letter are not significantly different at *α* = 5%. nd = not detected.

**Table 6:** Mean concentrations of *α*-pinene, terpinolene, and cannabidiol (CBD) obtained from the 2 materials and the 5 distillation times (DT).

Material	*α*-Pinene	Terpinolene	CBD	DT (min)	*α*-Pinene	Terpinolene	CBD
Ground	14.85 ^a^	4.41 ^a^	1.01 ^b^	0–5	14.70 ^b^	4.01 ^a^	nd
Nonground	12.70 ^b^	1.89 ^b^	1.11 ^a^	5–10	16.33 ^a^	3.35 ^b^	nd
				10–20	15.56 ^ab^	3.21 ^bc^	0.17 ^c^
				20–80	12.50 ^c^	2.84 ^c^	1.75 ^b^
				80–120	9.79 ^d^	2.34 ^d^	3.38 ^a^

Within each column, means followed by the same letter are not significantly different at *α* = 5%. nd = not detected.

**Table 7:** ANOVA *p*-values (*p*) that show the significance of the main and interaction effects of Material and distillation time (DT) on 9 antimicrobial activities. Significant effects that require multiple means comparison are shown in bold.

Source of Variation	SA	EF	PA	SP	YE	SE	CA	CK	CT
Material	**0.005**	0.068	0.002	0.427	0.522	0.940	0.170	0.082	0.002
DT	**0.001**	0.335	0.083	0.476	0.070	**0.001**	0.001	0.001	0.001
Material*DT	0.265	**0.043**	**0.012**	**0.001**	**0.004**	0.180	**0.001**	**0.001**	**0.001**

PA—*Pseudomonas aeroginosa* CCM 1959 (PA), SE—*Salmonella enterica subsp*. *enterica* CCM 3807, YE—*Yersinia enterocolitica* CCM 5671, EF—*Enterococcus faecalis* CCM 4224, SA—*Staphylococcus aureus* subs. *aureus* CCM 4223, SP—*Streptococcus pneumonia* CCM 4501, CA—*Candida albicans* CCM 8186, CK—C. *krusei* CCM 8271, CT—C. *tropicalis* CCM 8223.

**Table 8:** Mean values of antimicrobial activities against EF, SP, PA, YE, CA, CK, and CT obtained from the 12 combinations of Material and Distillation time (DT).

**Material**	**DT (min)**	**EF**	**SP**	**PA**	**YE**	**CA**	**CK**	**CT**
Grinded	0–5	5.83 ^ab^	5.00 ^ab^	3.17 ^abc^	2.50 ^b^	5.67 ^bc^	5.17 ^bc^	7.00 ^c^
Grinded	5–10	4.50 ^ab^	3.50 ^b^	3.50 ^abc^	4.00 ^ab^	6.00 ^bc^	4.00 ^bc^	13.8 ^a^
Grinded	10–20	5.83 ^ab^	5.33 ^ab^	2.83 ^bc^	2.00 ^b^	5.00 ^bc^	6.17 ^b^	7.67 ^bc^
Grinded	20–80	7.17 ^a^	7.17 ^a^	5.17 ^a^	3.00 ^ab^	5.33 ^bc^	3.50 ^bc^	5.00 ^c^
Grinded	80–120	2.83 ^b^	3.00 ^b^	3.00 ^abc^	3.00 ^ab^	5.00 ^bc^	3.17 ^c^	6.83 ^c^
Grinded	120–160	4.17 ^ab^	3.17 ^b^	1.67 ^c^	5.67 ^ab^	4.67 ^bc^	5.50 ^bc^	4.83 ^c^
Nongrinded	0–5	3.17 ^ab^	2.83 ^b^	3.00 ^abc^	1.83 ^b^	9.17 ^a^	12.00 ^a^	10.7 ^ab^
Nongrinded	5–10	5.00 ^ab^	2.67 ^b^	3.33 ^abc^	1.00 ^b^	7.33 ^ab^	5.33 ^bc^	7.67 ^bc^
Nongrinded	10–20	4.50 ^ab^	3.83 ^ab^	2.33 ^bc^	5.17 ^ab^	5.67 ^bc^	4.00 ^bc^	5.00 ^c^
Nongrinded	20–80	3.00 ^ab^	2.50 ^b^	1.67 ^c^	8.17 ^a^	4.50 ^bc^	3.00 ^c^	4.17 ^c^
Nongrinded	80–120	3.50 ^ab^	3.83 ^ab^	3.50 ^abc^	4.00 ^ab^	3.83 ^c^	3.00 ^c^	4.33 ^c^
Nongrinded	120–160	4.8 ^ab^	2.83 ^b^	4.17 ^ab^	2.67 ^ab^	4.00 ^bc^	3.83 ^bc^	4.33 ^c^
**Antibiotics**		**EF**	**SP**	**PA**	**YE**	**CA**	**CK**	**CT**
Gentamycin		24.3 ± 0.6	16.7 ±1.5	21.0 ± 1.0				
Cefoxitin					17.0 ± 2.00			
Fluconazole						18.3 ± 0.6	16.7 ± 1.5	17.3 ± 0.6

Within each column, means followed by the same letter are not significantly different at *α* = 5%. PA—*Pseudomonas aeroginosa* CCM 1959 (PA), SE—*Salmonella enterica* subsp. *enterica* CCM 3807, YE—*Yersinia enterocolitica* CCM 5671, EF—*Enterococcus faecalis* CCM 4224, SA—*Staphylococcus aureus* subs. *aureus* CCM 4223, SP—*Streptococcus pneumonia* CCM 4501, CA—*Candida albicans* CCM 8186, CK—*C. krusei* CCM 8271, CT—*C. tropicalis* CCM 8223.

**Table 9:** Mean values of antimicrobial activity of essential oils against SA obtained from the two Materials, and mean values of antimicrobial activities of essential oil fractions against SA and SE obtained from the 6 Distillation times (DT).

Material	SA	DT (min)	SA	SE
Grinded	5.72 ^a^	0–5	4.00 ^bc^	7.42 ^ab^
Nongrinded	4.50 ^b^	5–10	6.58 ^a^	4.33 ^b^
		10–20	4.58 ^abc^	9.92 ^a^
		20–80	6.25 ^a^	8.33 ^ab^
		80–120	6.00 ^ab^	5.92 ^b^
		120–160	3.25 ^c^	4.92 ^b^

Within each column, means followed by the same letter are not significantly different at *α* = 5%. SA—*Staphylococcus aureus* subs. *aureus* CCM 4223, SE—*Salmonella enterica* subsp. *enterica* CCM.

## Supplementary Materials

The following are available online. Table S1: Industrial Hemp essential oil composition of control oil and literature reports, Table S2: Essential oil constituents from nonground hemp material cv. Novosadska, Table S3: Essential oil constituents from ground hemp material cv. Novosadska.
