# Supplementary material for: Grinding and Fractionation during Distillation Alter Hemp Essential Oil Profile and Its Antimicrobial Activity
Source: Molecules. 2020 Aug 28;25(17):3943. doi: 10.3390/molecules25173943 (PMC7504750; doi:10.3390/molecules25173943)
Supplement: Supplementary file 1 [file molecules-25-03943-s001.pdf]

Grinding and Fractionation During Distillation Alter Hemp Essential Oil Profile and its Antimicrobial Activity

Valtcho D. Zheljazkov[1]*, Vladimir Sikora[2], Ivanka B. Semerdjieva[3], Miroslava Kačániová[4], Tess Astatkie[5], Ivayla Dincheva[6]

[1] Crop and Soil Science Department, 3050 SW Campus Way, Oregon State University, Corvallis, OR 97331, U.S.A. Valtcho.jeliazkov@oregonstate.edu

[2] Institute for Field and Vegetable Crops, Alternative Crops and Organic Production Department, Maksima Gorkog 30, 21000 Novi Sad, Serbia, vladimir.sikora@ifvcns.ns.ac.rs

[3]Department of Botany and Agrometeorology, Faculty of Agronomy, Agricultural University, Plovdiv, Bulgaria, v_semerdjieva@abv.bg

[4] Department of Fruit Science, Viticulture and Enology, Faculty of Horticulture and Landscape Engineering, Tr. A. Hlinku 2, Slovak University of Agriculture in Nitra, 949 76 Nitra, Slovak Republic

[4] Department of Bioenergetics and Food Analysis, Institution of Food Technology and Nutrition, University of Rzeszow, Cwiklinskiej 1, 35-601 Rzeszow, Poland; kacaniova.miroslava@gmail.com

[5] Department of Engineering, Faculty of Agriculture, Dalhousie University, Truro, NS, B2N 5E3, Canada astatkie@dal.ca

[6]Plant Genetic Research Group, Agrobioinstitute, Agricultural Academy, 8 "Dragan Tsankov" Blvd. 1164 Sofia, Bulgaria, ivadincheva@yahoo.com

* Correspondence: ValtchoJeliazkov@oregonstate.edu; Tel.: 1-541-737-5877)

Supplemental Table S1. Industrial Hemp essential oil composition of control oil.

RT	RI	Name	Rep 1	Rep 2
			Control 0-180 min	Control 0-180 min
		EO content, %	0.5	0.43
8.49	899	heptanal	0.136	0.144
9.14	918	2-methyl-4-heptanone	0.152	0.161
9.38	924	*α*-thujene	0.320	0.338
9.65	932	*α*-pinene	5.728	6.053
10.11	946	camphene	0.217	0.229
10.92	969	sabinene	0.154	0.163
11.03	974	*β*-pinene	3.075	3.250
11.52	988	myrcene	7.402	6.822
12.00	1002	*α*-phellandrene	0.078	0.082
12.10	1007	*δ*-3-carene	4.346	4.593
12.35	1014	*α*-terpinene	0.319	0.337
12.58	1020	*p*-cymene	0.144	0.152
12.78	1023	limonene	2.315	2.446
12.84	1026	eucalyptol	1.337	1.413
13.02	1032	*β*-(Z)-ocimene	1.743	1.842
13.41	1044	*β*-(E)-ocimene	8.345	8.819
13.72	1055	*γ*-terpinene	0.456	0.482
14.07	1066	(Z)-sabinene hydrate	0.361	0.382
14.59	1087	terpinolene	4.428	4.179
14.71	1090	*p*-cymenene	0.132	0.139
15.02	1098	(E)-sabinene hydrate	0.674	0.712
15.59	1114	endo-fenchol	0.137	0.145
15.78	1117	exo-fenchol	0.125	0.132
15.91	1128	allo-ocimene	0.246	0.260
16.27	1135	(E)-pinocarveol	0.262	0.277
16.33	1140	(Z)-pinene hydrate	0.141	0.149
16.89	1159	pinocarvone	0.153	0.162
17.20	1166	borneol	0.159	0.168
17.41	1171	(Z)-pinocamphone	0.164	0.173
17.52	1175	m-Cymen-8-ol	0.195	0.206
17.62	1180	*p*-cymen-8-ol	0.108	0.114
17.81	1188	*α*-terpineol	0.363	0.384
18.24	1205	verbenone	0.206	0.218
18.69	1290	(E)-verbenyl acetate	0.071	0.075
22.77	1372	*α*-ylangene	0.065	0.069
22.94	1376	*α*-copaene	0.049	0.052
23.15	1388	*β*-bourbonene	0.087	0.092
23.26	1393	7-epi-sesquithujene	0.102	0.108
23.71	1407	isocaryophyllene (*γ*-caryophyllene)	0.400	0.423
24.01	1411	*α*-(Z)-bergamotene	0.319	0.337
24.19	1418	*β*-caryophyllene	9.850	8.409
24.32	1430	*γ*-elemene	0.323	0.341
24.41	1432	*α*-(E)-bergamotene	2.216	2.342
24.60	1435	*β*-gurjunene	0.160	0.169
24.89	1441	(Z)-*β*-farnesene	2.335	2.468
25.05	1452	*α*-humulene (*α*-caryophyllene)	4.698	4.165
25.13	1457	*L*-alloaromadendrene	0.393	0.415
25.47	1478	*γ*-muurolene	0.155	0.164
25.52	1481	*γ*-himachalene	0.162	0.171
25.60	1485	germacrene D	0.087	0.092

25.70	1489	β-selinene	0.580	0.613
25.84	1492	δ-selinene	1.309	1.383
25.90	1497	α-selinene	0.324	0.342
26.01	1501	β-himachalene	0.945	0.999
26.16	1513	γ-cadinene	0.522	0.552
26.25	1522	δ-cadinene	0.641	0.677
26.44	1536	α-cadinene	0.336	0.355
26.57	1544	selina-3,7(11)-diene	0.377	0.398
26.68	1548	elemol	0.468	0.495
26.89	1566	maaliol	0.913	0.965
27.02	1570	caryophyllenyl alcohol	2.074	2.192
27.11	1572	germacrene D-4-ol	1.589	1.679
27.55	1576	spathulenol	2.312	2.443
27.68	1579	himachalene epoxide	0.115	0.122
28.12	1584	caryophyllene oxide	3.691	3.901
28.39	1590	globulol	0.313	0.331
28.45	1601	ledol	0.186	0.197
28.72	1608	humulene epoxide 2	1.557	1.645
28.83	1615	β-himachalene oxide	0.395	0.417
29.01	1626	selina-6-en-4-ol	1.092	1.154
29.25	1637	caryophylla-4(12),8(13)-dien-5α-ol	0.574	0.607
29.33	1639	caryophylla-4(12),8(13)-dien-5β-ol	0.728	0.769
29.53	1663	14-hydroxy-(Z)-caryophyllene	0.319	0.337
29.74	1668	14-hydroxy-9-epi-(E)-caryophyllene	0.901	0.952
29.95	1671	bulnesol	0.423	0.447
30.07	1675	β-bisabolol	1.047	1.106
30.40	1686	α-bisabolol	1.290	1.363
30.69	1694	eudesm-7(11)-en-4-ol	0.458	0.484
33.68	1822	(E)-nerolidyl isobutyrate	0.129	0.136
38.83	1943	phytol	0.136	0.144
40.64	2095	methyl linoleate	0.182	0.192
41.82	2132	linoleic acid	0.054	0.057
42.34	2283	δ9-tetrahydrocannabivarin	0.105	0.111
43.30	2306	cannabicyclol	0.091	0.096
44.11	2381	CBD	5.923	5.426
44.28	2399	cannabichromene	0.327	0.346
45.05	2462	δ8-tetrahydrocannabinol	0.126	0.133
45.65	2484	δ9-tetrahydrocannabinol (dronabinol)	0.138	0.146
		Monoterpenes	43.904	44.897264
		Sesquiterpenes	47.009	46.878594
		Cannabinoids	6.71	6.2576291
		Others(acid esters, ketone, alcohols)	0.66	0.697

Supplemental Table S2. Essential oil constituents from nonground hemp material cv. Novosadska.

RT	RI	Name	Control 0-180min	Control 0-180min	0-5min	0-5min	5-10min	5-10 min	10-20min	10-20min	20-80min	20-80min	80-120min	80-120 min	120-160min	120-160 min	160-180min
8.49	899	heptanal	0.136	0.144	0.196	0.188	nd	nd	nd	nd	nd	nd	nd	nd	nd	nd	nd
9.14	918	2-methyl-4-heptanone	0.152	0.161	0.378	0.362	0.208	0.220	0.167	0.160	0.103	0.109	nd	nd	nd	nd	nd
9.38	924	*α*-thujene	0.320	0.338	0.729	0.697	0.388	0.410	0.351	0.336	0.234	0.247	nd	nd	nd	nd	nd
9.65	932	*α*-pinene	5.728	6.053	14.128	13.518	15.494	15.184	14.602	13.971	11.490	12.143	8.022	8.478	6.155	6.889	5.134
10.11	946	camphene	0.217	0.229	0.540	0.517	0.303	0.320	0.234	0.224	0.138	0.146	nd	nd	nd	nd	nd
10.92	969	sabinene	0.154	0.163	0.216	0.207	0.164	0.173	0.119	0.114	nd	nd	nd	nd	nd	nd	nd
11.03	974	*β*-pinene	3.075	3.250	7.014	6.711	6.234	6.088	5.269	5.041	3.415	3.109	1.685	1.781	0.966	0.924	0.753
11.52	988	myrcene	7.402	6.822	15.126	15.472	14.739	13.869	14.529	13.901	10.912	10.532	7.741	8.181	5.290	5.606	3.438
12.00	1002	*α*-phellandrene	0.078	0.082	0.207	0.198	0.132	0.139	0.109	0.104	nd	nd	nd	nd	nd	nd	nd
12.10	1007	*δ*-3-carene	4.346	4.593	7.838	8.499	6.091	6.437	5.787	5.537	4.529	4.086	2.992	3.162	2.167	2.407	1.766
12.35	1014	*α*-terpinene	0.319	0.337	0.274	0.262	nd	nd	nd	nd	nd	nd	nd	nd	nd	nd	nd
12.58	1020	*p*-cymene	0.144	0.152	0.259	0.248	0.203	0.215	0.138	0.132	nd	nd	nd	nd	nd	nd	nd
12.78	1023	limonene	2.315	2.446	4.118	3.940	3.134	3.312	2.861	2.737	1.886	1.993	1.010	1.067	0.635	0.608	nd
12.84	1026	eucalyptol	1.337	1.413	2.651	2.536	1.024	1.082	nd	nd	nd	nd	nd	nd	nd	nd	nd
13.02	1032	*β*-(Z)-ocimene	1.743	1.842	2.743	2.624	1.725	1.823	1.513	1.448	1.346	1.422	0.641	0.677	nd	nd	nd
13.41	1044	*β*-(E)-ocimene	8.345	8.819	14.917	15.272	15.018	14.877	12.429	13.714	10.168	9.645	7.022	6.742	4.708	5.250	2.567
13.72	1055	*γ*-terpinene	0.456	0.482	0.477	0.456	0.236	0.249	0.215	0.206	0.173	0.183	nd	nd	nd	nd	nd
14.07	1066	(Z)-sabinene hydrate	0.361	0.382	0.427	0.409	0.260	0.275	nd	nd	nd	nd	nd	nd	nd	nd	nd
14.59	1087	terpinolene	4.428	4.179	3.078	2.945	1.977	2.089	1.942	1.858	1.553	1.641	0.878	0.928	0.533	0.510	nd
14.71	1090	*p*-cymenene	0.132	0.139	0.140	0.128	nd	nd	nd	nd	nd	nd	nd	nd	nd	nd	nd
15.02	1098	(E)-sabinene hydrate	0.674	0.712	0.789	0.755	0.513	0.542	0.189	0.181	nd	nd	nd	nd	nd	nd	nd
15.59	1114	endo-fenchol	0.137	0.145	0.181	0.173	0.160	0.169	nd	nd	nd	nd	nd	nd	nd	nd	nd
15.78	1117	exo-fenchol	0.125	0.132	0.148	0.142	nd	nd	nd	nd	nd	nd	nd	nd	nd	nd	nd
15.91	1128	allo-ocimene	0.246	0.260	0.237	0.227	nd	nd	nd	nd	nd	nd	nd	nd	nd	nd	nd
16.27	1135	(E)-pinocarveol	0.262	0.277	0.112	0.107	nd	nd	nd	nd	nd	nd	nd	nd	nd	nd	nd
16.33	1140	(Z)-pinene hydrate	0.141	0.149	0.101	0.097	nd	nd	nd	nd	nd	nd	nd	nd	nd	nd	nd
16.89	1159	pinocarvone	0.153	0.162	0.122	0.117	nd	nd	nd	nd	nd	nd	nd	nd	nd	nd	nd
17.20	1166	borneol	0.159	0.168	0.078	0.075	nd	nd	nd	nd	nd	nd	nd	nd	nd	nd	nd

17.41	1171	(Z)-pinocamphone	0.164	0.173	0.118	0.113	0.152	0.161	nd	nd	nd	nd	nd	nd	nd	nd	nd
17.52	1175	*m*-cymen-8-ol	0.195	0.206	nd	nd	nd	nd	nd	nd	nd	nd	nd	nd	nd	nd	nd
17.62	1180	*p*-cymen-8-ol	0.108	0.114	nd	nd	nd	nd	nd	nd	nd	nd	nd	nd	nd	nd	nd
17.81	1188	*α*-terpineol	0.363	0.384	0.165	0.158	0.210	0.222	0.202	0.193	nd	nd	nd	nd	nd	nd	nd
18.24	1205	verbenone	0.206	0.218	nd	nd	nd	nd	nd	nd	nd	nd	nd	nd	nd	nd	nd
18.69	1290	(E)-verbenyl acetate	0.071	0.075	nd	nd	nd	nd	nd	nd	nd	nd	nd	nd	nd	nd	nd
22.77	1372	*α*-ylangene	0.065	0.069	nd	nd	nd	nd	nd	nd	nd	nd	nd	nd	nd	nd	nd
22.94	1376	*α*-copaene	0.049	0.052	nd	nd	nd	nd	nd	nd	nd	nd	nd	nd	nd	nd	nd
23.15	1388	*β*-bourbonene	0.087	0.092	nd	nd	nd	nd	nd	nd	nd	nd	nd	nd	nd	nd	nd
23.26	1393	7-epi-sesquithujene	0.102	0.108	nd	nd	nd	nd	nd	nd	nd	nd	nd	nd	nd	nd	nd
23.71	1407	isocaryophyllene (*γ*-caryophyllene)	0.400	0.423	0.222	0.212	0.171	0.181	0.183	0.175	0.250	0.264	0.323	0.341	0.363	0.347	nd
24.01	1411	*α*-(Z)-bergamotene	0.319	0.337	0.147	0.141	nd	nd	0.106	0.101	0.145	0.153	0.189	0.200	nd	nd	nd
24.19	1418	*β*-caryophyllene	9.850	8.409	8.342	8.938	11.403	12.051	11.815	12.430	14.206	13.601	21.041	20.236	25.399	24.826	37.677
24.32	1430	*γ*-elemene	0.323	0.341	0.112	0.107	0.138	0.146	0.179	0.171	0.271	0.286	0.363	0.384	0.403	0.386	nd
24.41	1432	*α*-(E)-bergamotene	2.216	2.342	1.350	1.292	1.112	1.175	1.099	1.052	1.453	1.536	1.981	2.093	2.363	2.261	3.135
24.60	1435	*β*-gurjunene	0.160	0.169	nd	nd	nd	nd	nd	nd	nd	nd	nd	nd	nd	nd	nd
24.89	1441	(Z)-*β*-farnesene	2.335	2.468	1.217	1.164	1.071	1.132	1.075	1.029	1.382	1.460	1.697	1.793	1.895	1.813	1.435
25.05	1452	*α*-humulene (*α*-caryophyllene)	4.698	4.165	3.006	3.259	4.221	3.846	4.521	5.033	5.710	6.034	7.773	7.021	9.058	8.667	11.250
25.13	1457	*L*-alloaromadendrene	0.393	0.415	0.178	0.170	0.185	0.196	0.195	0.187	0.261	0.276	0.366	0.387	0.442	0.423	nd
25.47	1478	*γ*-muurolene	0.155	0.164	nd	nd	nd	nd	nd	nd	nd	nd	nd	nd	nd	nd	nd
25.52	1481	*γ*-himachalene	0.162	0.171	nd	nd	nd	nd	nd	nd	nd	nd	nd	nd	nd	nd	nd
25.60	1485	germacrene D	0.087	0.092	nd	nd	nd	nd	nd	nd	nd	nd	nd	nd	nd	nd	nd
25.70	1489	*β*-selinene	0.580	0.613	0.288	0.276	0.280	0.296	0.318	0.304	0.430	0.454	0.548	0.579	0.614	0.587	nd
25.84	1492	*δ*-selinene	1.309	1.383	0.683	0.653	0.732	0.774	0.784	0.750	1.037	1.096	1.283	1.036	1.496	1.431	1.711
25.90	1497	*α*-selinene	0.324	0.342	0.146	0.140	nd	nd	0.186	0.178	0.253	0.267	0.293	0.310	nd	nd	nd
26.01	1501	*β*-himachalene	0.945	0.999	0.486	0.465	0.510	0.539	0.577	0.552	0.751	0.794	0.912	0.964	1.051	1.006	1.282
26.16	1513	*γ*-cadinene	0.522	0.552	0.224	0.214	0.176	0.186	0.241	0.231	0.327	0.346	0.320	0.338	0.364	0.348	nd
26.25	1522	*δ*-cadinene	0.641	0.677	0.279	0.267	0.231	0.244	0.315	0.301	0.419	0.443	0.416	0.440	0.482	0.461	nd
26.44	1536	*α*-cadinene	0.336	0.355	0.169	0.162	nd	nd	0.155	0.148	0.202	0.213	nd	nd	nd	nd	nd
26.57	1544	selina-3,7(11)-diene	0.377	0.398	0.196	0.188	0.154	0.163	0.200	0.191	0.346	0.366	0.312	0.330	0.358	0.343	nd
26.68	1548	elemol	0.468	0.495	0.137	0.131	nd	nd	0.229	0.219	0.296	0.313	nd	nd	nd	nd	nd
26.89	1566	maaliol	0.913	0.965	0.312	0.299	0.337	0.356	0.369	0.353	0.488	0.516	0.620	0.655	0.729	0.697	0.812
27.02	1570	caryophyllenyl alcohol	2.074	2.192	0.883	0.845	1.141	1.206	1.303	1.247	1.920	2.029	2.329	2.046	2.677	2.561	3.147
27.11	1572	germacrene D-4-ol	1.589	1.679	0.738	0.706	0.976	1.031	1.093	1.046	1.470	1.553	2.024	2.139	2.411	2.307	3.265
27.55	1576	spathulenol	2.312	2.443	0.604	0.578	1.140	1.205	1.694	1.621	2.430	2.568	2.813	2.497	2.875	2.751	2.834
27.68	1579	himachalene epoxide	0.115	0.122	nd	nd	nd	nd	nd	nd	nd	nd	nd	nd	nd	nd	nd

28.12	1584	caryophyllene oxide	3.691	3.901	1.101	1.053	3.735	3.254	5.133	5.591	6.576	6.146	7.447	7.870	6.985	7.468	6.606
28.39	1590	globulol	0.313	0.331	nd	nd	nd	nd	nd	nd	nd	nd	nd	nd	nd	nd	nd
28.45	1601	ledol	0.186	0.197	nd	nd	nd	nd	0.158	0.151	0.248	0.262	0.242	0.256	nd	nd	nd
28.72	1608	humulene epoxide 2	1.557	1.645	0.259	0.248	0.919	0.971	1.535	1.469	2.078	2.407	2.544	2.169	2.432	2.327	2.422
28.83	1615	*β*-himachalene oxide	0.395	0.417	nd	nd	nd	nd	0.298	0.285	0.486	0.514	0.516	0.545	nd	nd	nd
29.01	1626	selina-6-en-4-ol	1.092	1.154	nd	nd	0.307	0.324	0.687	0.657	1.062	1.122	1.377	1.455	1.444	1.382	nd
29.25	1637	caryophylla-4(12),8(13)-dien-5α-ol	0.574	0.607	nd	nd	nd	nd	0.481	0.460	0.659	0.696	0.967	1.022	0.800	0.765	nd
29.33	1639	caryophylla-4(12),8(13)-dien-5β-ol	0.728	0.769	nd	nd	0.298	0.315	0.381	0.365	1.244	1.315	0.827	0.874	0.862	0.825	nd
29.53	1663	14-hydroxy-(Z)-caryophyllene	0.319	0.337	nd	nd	nd	nd	0.202	0.193	0.374	0.395	0.753	0.796	0.900	0.861	nd
29.74	1668	14-hydroxy-9-epi-(E)-caryophyllene	0.901	0.952	nd	nd	0.150	0.159	0.436	0.417	0.782	0.826	0.926	0.979	0.936	0.896	nd
29.95	1671	bulnesol	0.423	0.447	nd	nd	nd	nd	nd	nd	0.195	0.206	0.228	0.241	nd	nd	nd
30.07	1675	*β*-bisabolol	1.047	1.106	nd	nd	0.215	0.227	0.475	0.454	1.014	1.072	1.365	1.443	1.527	1.461	2.116
30.40	1686	*α*-bisabolol	1.290	1.363	nd	nd	0.318	0.336	0.863	0.826	1.751	1.585	2.308	2.044	2.603	2.491	2.502
30.69	1694	eudesm-7(11)-en-4-ol	0.458	0.484	nd	nd	nd	nd	0.164	0.157	0.364	0.385	0.460	0.486	0.539	0.516	nd
33.68	1822	(E)-nerolidyl isobutyrate	0.129	0.136	nd	nd	nd	nd	nd	nd	nd	nd	nd	nd	0.133	0.141	nd
38.83	1943	phytol	0.136	0.144	nd	nd	nd	nd	nd	nd	nd	nd	nd	nd	0.115	0.108	nd
40.64	2095	methyl linoleate	0.182	0.192	nd	nd	nd	nd	nd	nd	nd	nd	nd	nd	0.165	0.152	nd
41.82	2132	linoleic acid	0.054	0.057	nd	nd	nd	nd	nd	nd	nd	nd	nd	nd	0.043	0.047	nd
42.34	2283	*δ*9-tetrahydrocannabivarin	0.105	0.111	nd	nd	nd	nd	nd	nd	nd	nd	nd	nd	0.089	0.093	nd
43.30	2306	cannabicyclol	0.091	0.096	nd	nd	nd	nd	nd	nd	nd	nd	nd	nd	0.077	0.072	nd
44.11	2381	CBD	5.923	5.426	nd	nd	nd	nd	0.345	0.318	1.675	1.844	3.351	3.541	5.137	5.271	4.696
44.28	2399	Cannabichromene	0.327	0.346	nd	nd	nd	nd	nd	nd	nd	nd	nd	nd	0.312	0.305	nd
45.05	2462	*δ*8-tetrahydrocannabinol	0.126	0.133	nd	nd	nd	nd	nd	nd	nd	nd	nd	nd	0.116	0.122	nd
45.65	2484	*δ*9-tetrahydrocannabinol (Dronabinol)	0.138	0.146	nd	nd	nd	nd	nd	nd	nd	nd	nd	nd	0.102	0.111	nd

Monoterpenes	43.904	44.89726	76.933	76.6027	68.157	67.63604	60.4888	59.69709	45.844	45.14743	29.991	31.01546	20.454	22.19476	13.658
Sesquiterpenes	47.009	46.87859	21.079	21.50766	29.92	30.31141	37.45	38.34458	50.8802	51.50048	65.563	63.96761	72.141	70.34695	80.1942
Cannabinoids	6.71	6.257629	0	0	0	0	0.345	0.318	1.6745	1.844097	3.351	3.5413	5.83318	5.97392	4.696
Others(acid esters, ketone, alcohol)	0.66	0.697	0.574	0.549197	0.208	0.219812	0.167	0.159784	0.103	0.108849	0	0	0.323	0.307	0

Supplemental Table S3. Essential oil constituents from ground hemp material cv. Novosadska.

RT	RI	Name	840	841	842	843	844	845	846	847	848	849	850	851
			0-5min	0-5min	5-10min	5-10 min	10-20min	10-20min	20-80min	20-80min	80-120min	80-120 min	120-180min	120-180 min
8.49	899	heptanal	0.242	0.232	0.191	0.202	0.177	0.169	nd	nd	nd	nd	nd	nd
9.14	918	2-methyl-4-heptanone	0.336	0.321	0.304	0.321	0.278	0.266	0.225	0.238	0.126	0.121	0.104	0.109
9.38	924	*α*-thujene	0.466	0.446	0.410	0.433	0.418	0.400	0.390	0.412	0.242	0.232	0.286	0.299
9.65	932	*α*-pinene	15.850	15.317	17.806	16.817	16.695	16.974	12.818	13.546	11.189	11.457	14.830	14.495
10.11	946	camphene	0.405	0.387	0.378	0.399	0.352	0.337	0.276	0.292	0.153	0.146	0.177	0.185
10.92	969	sabinene	0.183	0.175	0.196	0.207	0.222	0.212	0.228	0.241	0.150	0.144	0.138	0.144
11.03	974	*β*-pinene	7.728	7.394	7.835	8.280	7.078	6.772	5.511	5.824	3.721	3.956	4.166	4.354
11.52	988	myrcene	19.077	18.525	21.078	20.275	18.296	18.505	14.232	13.704	12.559	12.890	12.980	12.521
12.00	1002	*α*-phellandrene	0.246	0.235	nd	nd	0.063	0.060	0.173	0.183	0.106	0.101	0.090	0.094
12.10	1007	*δ*-3-carene	5.502	5.826	5.173	5.467	4.821	5.361	4.449	4.702	3.367	3.722	5.558	5.809
12.35	1014	*α*-terpinene	0.225	0.215	nd	nd	0.213	0.204	0.216	0.228	0.129	0.123	nd	nd
12.58	1020	*p*-cymene	0.284	0.272	0.293	0.310	0.220	0.210	0.121	0.128	0.098	0.094	0.103	0.108
12.78	1023	limonene	3.107	2.973	2.652	2.280	2.527	2.842	2.591	2.738	1.896	1.814	2.403	2.118
12.84	1026	eucalyptol	5.470	5.823	4.076	4.307	2.382	2.628	0.814	0.860	0.230	0.243	0.204	0.213
13.02	1032	*β*-(Z)-ocimene	2.234	2.137	1.996	2.109	2.168	2.074	4.138	3.737	1.276	1.221	0.962	1.005
13.41	1044	*β*-(E)-ocimene	15.639	16.319	17.004	17.603	15.775	16.105	13.451	12.921	12.329	12.580	9.982	10.433
13.72	1055	*γ*-terpinene	0.398	0.381	0.270	0.285	0.321	0.307	0.382	0.404	0.232	0.222	0.244	0.255
14.07	1066	(Z)-sabinene hydrate	0.634	0.607	0.494	0.522	0.378	0.362	0.280	0.296	nd	nd	nd	nd
14.59	1087	terpinolene	5.125	4.904	4.533	4.790	4.610	4.411	4.449	3.702	3.687	3.853	2.843	2.536
14.71	1090	*p*-cymenene	nd	nd	nd	nd	nd	nd	nd	nd	nd	nd	nd	nd
15.02	1098	(E)-sabinene hydrate	0.755	0.722	0.625	0.660	0.576	0.551	0.481	0.508	nd	nd	nd	nd
15.59	1114	endo-fenchol	0.199	0.190	nd	nd	0.120	0.115	0.089	0.094	nd	nd	nd	nd
15.78	1117	exo-fenchol	nd	nd	nd	nd	0.112	0.107	0.092	0.097	nd	nd	nd	nd
15.91	1128	allo-ocimene	nd	nd	nd	nd	nd	nd	0.116	0.123	nd	nd	nd	nd
16.27	1135	(E)-pinocarveol	0.156	0.149	nd	nd	nd	nd	0.054	0.057	nd	nd	nd	nd
16.33	1140	(Z)-pinene hydrate	nd	nd	nd	nd	nd	nd	0.213	0.225	nd	nd	nd	nd
16.89	1159	pinocarvone	nd	nd	nd	nd	nd	nd	0.097	0.103	nd	nd	nd	nd
17.20	1166	borneol	nd	nd	nd	nd	0.145	0.139	0.155	0.164	nd	nd	nd	nd
17.41	1171	(Z)-pinocamphone	0.162	0.155	nd	nd	0.161	0.154	0.141	0.149	nd	nd	nd	nd

17.52	1175	*m*-cymen-8-ol	nd	nd	nd	nd	0.145	0.152	0.202	0.213	nd	nd	nd	nd
17.62	1180	*p*-cymen-8-ol	nd	nd	nd	nd	0.111	0.120	0.089	0.095	nd	nd	nd	nd
17.81	1188	*α*-terpineol	0.147	0.141	nd	nd	0.170	0.163	0.375	0.396	nd	nd	nd	nd
18.24	1205	verbenone	nd	nd	nd	nd	nd	nd	0.193	0.204	nd	nd	nd	nd
18.69	1290	(E)-verbenyl acetate	nd	nd	nd	nd	nd	nd	0.128	0.135	nd	nd	nd	nd
22.77	1372	*α*-ylangene	nd	nd	nd	nd	nd	nd	0.061	0.067	nd	nd	nd	nd
22.94	1376	*α*-copaene	nd	nd	nd	nd	nd	nd	0.055	0.059	nd	nd	nd	nd
23.15	1388	*β*-bourbonene	nd	nd	nd	nd	nd	nd	0.086	0.090	nd	nd	nd	nd
23.26	1393	7-epi-sesquithujene	nd	nd	nd	nd	nd	nd	0.074	0.079	nd	nd	nd	nd
23.71	1407	isocaryophyllene (*γ*-caryophyllene)	nd	nd	nd	nd	0.082	0.078	0.120	0.127	0.191	0.183	0.169	0.177
24.01	1411	*α*-(Z)-bergamotene	nd	nd	nd	nd	nd	nd	0.071	0.075	0.117	0.112	nd	nd
24.19	1418	*β*-caryophyllene	6.175	6.454	5.872	5.421	6.467	6.188	7.694	6.913	10.850	10.295	12.836	12.371
24.32	1430	*γ*-elemene	nd	nd	nd	nd	0.142	0.136	0.201	0.212	0.285	0.273	0.178	0.186
24.41	1432	*α*-(E)-bergamotene	0.699	0.669	0.496	0.524	0.525	0.502	0.690	0.729	1.142	1.093	0.920	0.962
24.60	1435	*β*-gurjunene	nd	nd	nd	nd	nd	nd	0.073	0.077	0.095	0.091	nd	nd
24.89	1441	(Z)-*β*-farnesene	0.579	0.554	0.281	0.297	0.438	0.419	0.676	0.714	1.037	0.992	0.635	0.664
25.05	1452	*α*-humulene (*α*-caryophyllene)	2.014	2.493	1.873	1.979	2.469	2.171	2.871	3.034	4.266	4.082	3.957	4.136
25.13	1457	*L*-alloaromadendrene	0.141	0.135	nd	nd	0.170	0.163	0.223	0.236	0.339	0.324	0.295	0.308
25.47	1478	*γ*-muurolene	nd	nd	nd	nd	nd	nd	0.108	0.114	0.071	0.068	nd	nd
25.52	1481	*γ*-himachalene	nd	nd	nd	nd	nd	nd	nd	nd	0.066	0.063	nd	nd
25.60	1485	germacrene D	nd	nd	nd	nd	nd	nd	nd	nd	0.085	0.092	nd	nd
25.70	1489	*β*-selinene	0.088	0.084	nd	nd	0.136	0.130	0.217	0.229	0.296	0.290	0.189	0.198
25.84	1492	*δ*-selinene	0.304	0.291	0.260	0.275	0.402	0.385	0.572	0.604	0.844	0.808	0.616	0.644
25.90	1497	*α*-selinene	nd	nd	nd	nd	0.080	0.077	0.122	0.129	0.167	0.160	nd	nd
26.01	1501	*β*-himachalene	0.246	0.235	nd	nd	0.312	0.299	0.443	0.468	0.655	0.627	0.453	0.473
26.16	1513	*γ*-cadinene	nd	nd	nd	nd	0.083	0.079	0.155	0.164	0.201	0.192	nd	nd
26.25	1522	*δ*-cadinene	nd	nd	nd	nd	0.140	0.134	0.229	0.242	0.321	0.307	nd	nd
26.44	1536	*α*-cadinene	nd	nd	nd	nd	0.097	0.093	0.139	0.147	0.194	0.186	nd	nd
26.57	1544	selina-3,7(11)-diene	nd	nd	nd	nd	0.106	0.101	0.152	0.161	0.221	0.211	0.189	0.198
26.68	1548	elemol	nd	nd	nd	nd	0.114	0.109	0.201	0.212	0.242	0.232	0.166	0.173
26.89	1566	maaliol	0.202	0.193	nd	nd	0.236	0.226	0.370	0.391	0.498	0.476	0.265	0.277
27.02	1570	caryophyllenyl alcohol	0.566	0.542	0.535	0.565	0.651	0.623	1.015	1.073	1.522	1.456	0.924	0.966
27.11	1572	germacrene D-4-ol	0.573	0.548	0.607	0.641	0.697	0.667	0.915	0.967	1.379	1.319	0.899	0.940
27.55	1576	spathulenol	0.655	0.627	0.787	0.832	0.976	0.934	1.614	1.706	2.127	2.350	1.272	1.329
27.68	1579	himachalene epoxide	nd	nd	nd	nd	nd	nd	nd	nd	0.135	0.129	0.140	0.142
28.12	1584	caryophyllene oxide	1.248	1.494	nd	nd	2.881	2.757	4.068	4.383	5.529	5.829	5.281	5.052

28.39	1590	globulol	nd	nd	2.013	2.127	0.105	0.100	0.227	0.240	0.257	0.246	nd	nd
28.45	1601	ledol	nd	nd	nd	nd	0.094	0.090	0.166	0.175	0.191	0.183	0.165	0.172
28.72	1608	humulene epoxide 2	0.258	0.247	0.447	0.472	0.761	0.728	1.222	1.291	1.673	1.601	1.700	1.777
28.83	1615	*β*-himachalene oxide	nd	nd	nd	nd	0.140	0.134	0.289	0.305	0.364	0.348	0.333	0.348
29.01	1626	selina-6-en-4-ol	nd	nd	nd	nd	0.413	0.395	0.780	0.824	1.093	1.046	0.897	0.937
29.25	1637	caryophylla-4(12),8(13)-dien-5α-ol	nd	nd	nd	nd	0.214	0.205	0.404	0.427	0.602	0.576	0.543	0.568
29.33	1639	caryophylla-4(12),8(13)-dien-5β-ol	nd	nd	nd	nd	0.121	0.116	0.525	0.555	0.879	0.841	1.098	1.148
29.53	1663	14-hydroxy-(Z)-caryophyllene	nd	nd	nd	nd	0.147	0.141	0.216	0.228	0.267	0.255	0.232	0.242
29.74	1668	14-hydroxy-9-epi-(E)-caryophyllene	nd	nd	nd	nd	0.146	0.140	0.556	0.519	0.814	0.779	0.587	0.613
29.95	1671	bulnesol	nd	nd	nd	nd	0.066	0.063	0.255	0.269	0.336	0.321	0.213	0.223
30.07	1675	*β*-bisabolol	nd	nd	nd	nd	0.094	0.090	0.528	0.558	0.845	0.808	1.043	1.090
30.40	1686	*α*-bisabolol	nd	nd	nd	nd	0.492	0.471	1.197	1.265	1.683	1.610	1.044	1.091
30.69	1694	eudesm-7(11)-en-4-ol	nd	nd	nd	nd	0.101	0.097	0.264	0.279	0.386	0.369	0.263	0.275
33.68	1822	(E)-nerolidyl isobutyrate	nd	nd	nd	nd	nd	nd	nd	nd	0.108	0.116	nd	nd
38.83	1943	phytol	nd	nd	nd	nd	nd	nd	nd	nd	0.122	0.131	nd	nd
40.64	2095	methyl linoleate	nd	nd	nd	nd	nd	nd	nd	nd	0.111	0.106	nd	nd
41.82	2132	linoleic acid	nd	nd	nd	nd	nd	nd	nd	nd	0.049	0.056	nd	nd
42.34	2283	*δ*9-tetrahydrocannabivarin	nd	nd	nd	nd	nd	nd	nd	nd	0.075	0.068	nd	nd
43.30	2306	cannabicyclol	nd	nd	nd	nd	nd	nd	nd	nd	0.070	0.061	nd	nd
44.11	2381	CBD	nd	nd	nd	nd	nd	nd	1.692	1.788	3.495	3.148	5.859	6.124
44.28	2399	cannabichromene	nd	nd	nd	nd	nd	nd	nd	nd	0.244	0.235	nd	nd
45.05	2462	*δ*8-Tetrahydrocannabinol	nd	nd	nd	nd	nd	nd	nd	nd	0.088	0.790	nd	nd
45.65	2484	*δ*9-Tetrahydrocannabinol (Dronabinol)	nd	nd	nd	nd	nd	nd	nd	nd	0.101	0.089	nd	nd

Monoterpenes	83.992	83.2945062	84.819	84.746445	78.079	79.2652561	66.944	66.4813541	51.3643796	52.7977541	54.9647728	54.5686
Sesquiterpenes	13.7476	14.5659859	13.171	13.1340494	20.098	19.0381875	29.844	30.3394514	42.373	41.3394554	37.5027659	37.6799
Cannabinoids	0	0	0	0	0	0	1.692	1.78808699	4.073	4.39128675	5.85937584	6.124
Others(acid esters, ketone, alcohol)	0.578	0.55302404	0.495	0.52311056	0.455	0.435339	0.225	0.23777753	0.408	0.414	0.10429	0.109
